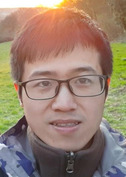# Germacrene A–A Central Intermediate in Sesquiterpene Biosynthesis

**DOI:** 10.1002/chem.202002163

**Published:** 2020-09-30

**Authors:** Houchao Xu, Jeroen S. Dickschat

**Affiliations:** ^1^ Kekulé-Institute for Organic Chemistry and Biochemistry University of Bonn Gerhard-Domagk-Straße 1 53121 Bonn Germany

**Keywords:** biosynthesis, configuration determination, enzyme catalysis, germacrene A, sesquiterpenes

## Abstract

This review summarises known sesquiterpenes whose biosyntheses proceed through the intermediate germacrene A. First, the occurrence and biosynthesis of germacrene A in Nature and its peculiar chemistry will be highlighted, followed by a discussion of 6–6 and 5–7 bicyclic compounds and their more complex derivatives. For each compound the absolute configuration, if it is known, and the reasoning for its assignment is presented.

## Graphical abstract

**Inspired by nature**: This review discusses the unique chemistry of germacrene A, its occurrence in Nature and its central role as an intermediate in the biosynthesis of sesquiterpenes. Biosynthetic derivatives include eudesmanes and guaianes and further compounds arising from skeletal rearrangements. For each compound the reasoning for its assigned absolute configuration is discussed, if it has been established, and the natural sources are summarised.

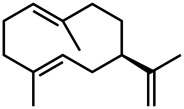

## 1 Introduction

**Scheme 1:**
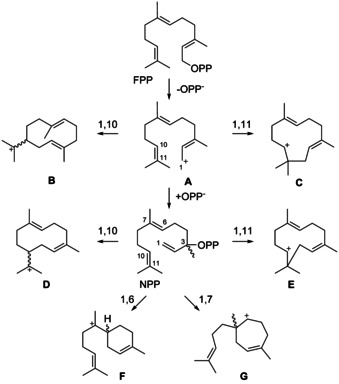
Terpene cyclisation modes for FPP.

With an estimated number of over 80,000 compounds terpenes form the largest class of natural products. They are produced by all kingdoms of life and can be classified as mono‐ (C_10_), sesqui‐ (C_15_) or diterpenes (C_20_) etc. according to the number of incorporated isoprenoid units. During the past decades many sesquiterpene synthases have been reported[^1^, ^2^, ^3^, ^4^, ^5^, ^6^] that catalyse the cyclisation of farnesyl diphosphate (FPP) through diphosphate abstraction to give the reactive farnesyl cation (**A**, Scheme 1). Attack of the C10=C11 double bond to C1 can yield the (*E*,*E*)‐germacradienyl cation (**B**) by 1,10‐ or the (*E*,*E*)‐humulyl cation (**C**) by 1,11‐cyclisation. The alternative reaction by reattack of diphosphate to C3 results in nerolidyl diphosphate (NPP). After a conformational rearrangement of the vinyl group by rotation around the C2−C3 bond, cyclisation reactions may proceed to the (*E*,*Z*)‐germacradienyl cation (**D**), the (*E*,*Z*)‐humulyl cation (**E**), the bisabolyl cation (**F**), or to cation **G**, with possible formation of either enantiomer for chiral intermediates. Deprotonation of **B** leads to germacrene A, a widespread natural product and central intermediate in the biosynthesis of many 1,10‐cyclised sesquiterpenes. This review discusses its occurrence in Nature, its chemistry, and central importance as an intermediate towards many sesquiterpenes.

## 2 Germacrene A

### 2.1 Occurrence in Nature

**Scheme 2:**
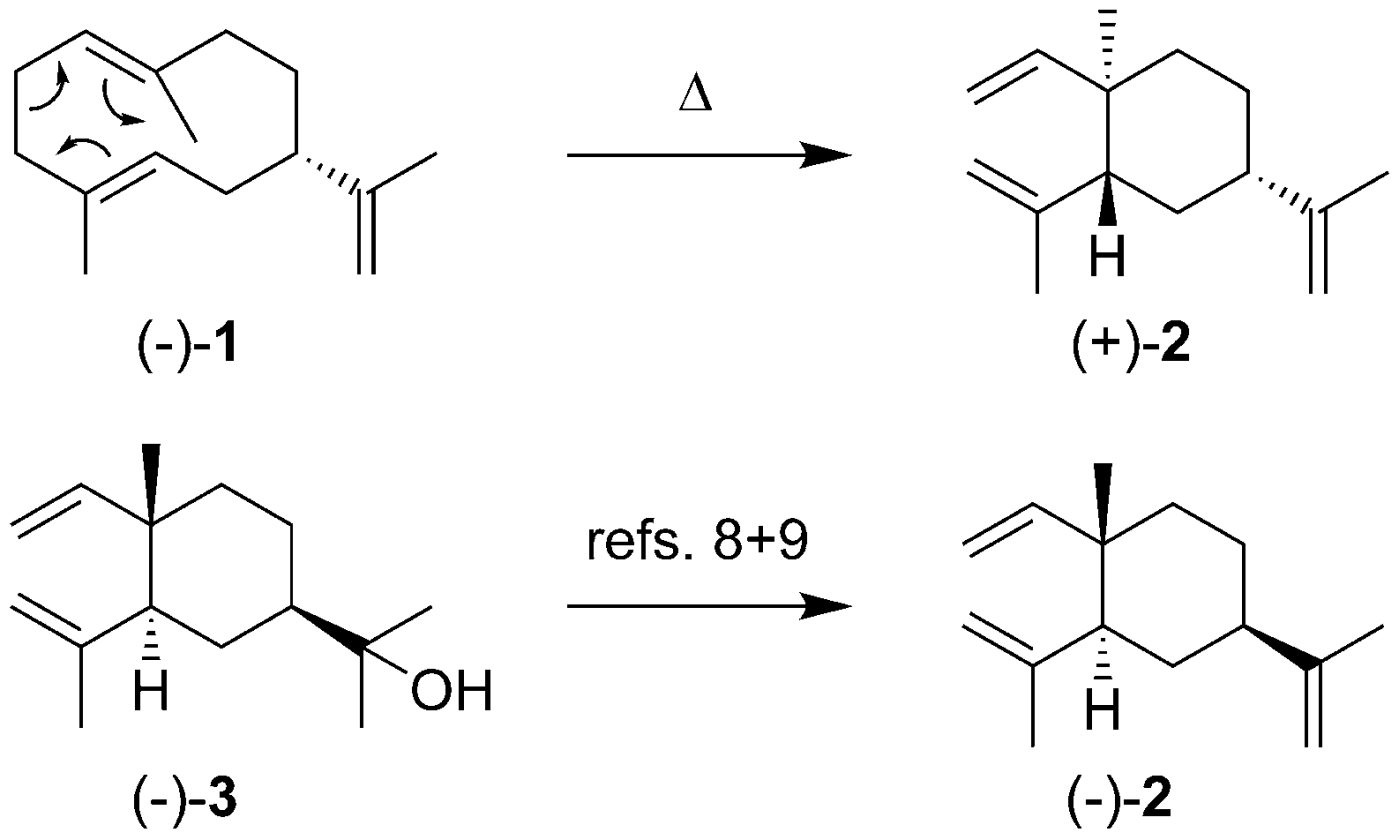
Structure of **1** and its absolute configuration by chemical correlation.

(−)‐Germacrene A (**1**, Scheme 2) was first isolated in 1970 from the gorgonian *Eunicea mammosa*.^[7]^ Its absolute configuration was established as (*S*)‐(−)‐**1** through its Cope rearrangement to (+)‐β‐elemene (**2**) for which the configurational assignment was performed by chemical correlation of (−)‐elemol (**3**) to (−)‐**2**.[^8^, ^9^] Compound (−)‐**1** is also believed to occur in the soft coral *Lobophytum*,^[10]^ and is the alarm pheromone of the aphid *Terioaphis maculata*.[^11^, ^12^] In the course of this work it was noticed that the optical rotation ([α]_D_^25^=−26.8, *c* 1.0, CCl_4_) was significantly higher than initially reported ([α]_D_^25^=−3.2, *c* 14.4, CCl_4_),^[7]^ which is explainable by a partial rearrangement of purified (−)‐**1** to (+)‐**2**, or alternatively, **1** isolated from *E. mammosa* was not enantiomerically pure. However, the optical rotation of (+)‐**2** ([α]_D_^25^=+15.1, neat) reported in this initial study^[7]^ matches the reported value for (−)‐**2** ([α]_D_^25^=−15.8, *c* 0.50, CHCl_3_) obtained by Cope rearrangement of (+)‐**1**,^[13]^ thus disfavouring the latter hypothesis. In fact, the enantiomeric composition of a compound cannot be concluded only from the optical rotation upon its first isolation, or not with certainty if a compound is known to be instable. Methods such as chromatographic separation on a chiral stationary phase may be more conclusive. Through this approach, König and co‐workers found that **1** from various plants is a mixture of enantiomers, ranging from nearly pure (+)‐**1** in *Piper nigrum* to mainly (−)‐**1** in the liverwort *Barbilophozia barbata*.^[14]^

**Scheme 3:**
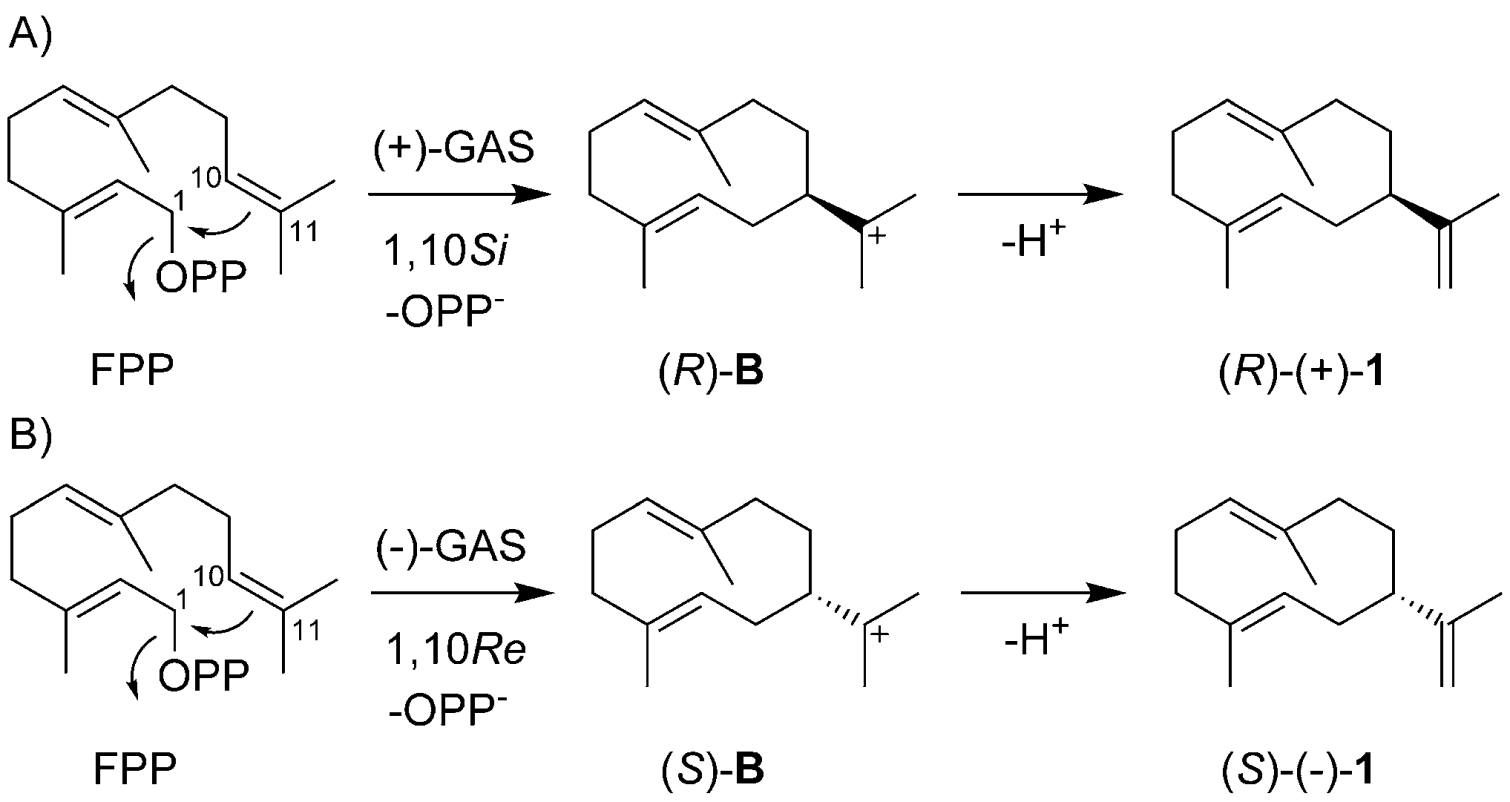
Cyclisation mechanism from FPP to A) (*R*)‐(+)‐**1** and B) (*S*)‐(−)‐**1**.

Germacrene A synthase (GAS) catalyses a 1,10‐cyclisation of FPP to **B**, followed by deprotonation to **1** (Scheme 3). Both enantiomers of **1** are accessible through this reaction, depending on whether C10 of FPP is attacked from the *Re* or the *Si* face. Since this face selectivity may be altered by subtle conformational changes of FPP in the active sites of GASs, predictions based on amino acid sequences or phylogenetic analyses regarding the stereochemical implications may be difficult. Many plant GAS have been identified during the past two decades, including two (+)‐GASs from *Cichorium intybus*[^15^, ^16^] and one from *Matricaria recutita*,^[17]^ with the absolute configuration of (+)‐**1** established by chiral GC. Sometimes the absolute configuration can be rationally suggested, because **1** is transformed in the same organism into another compound such as (+)‐costunolide.[^18^, ^19^, ^20^, ^21^] Further GASs are known from many other plant species,[^22^, ^23^, ^24^, ^25^, ^26^, ^27^, ^28^, ^29^, ^30^, ^31^, ^32^] but the absolute configuration of **1** has frequently not been determined. While the accumulated literature shows that (+)‐**1** is typical for plants, the recently characterised bacterial GAS from *Micromonospora marina* produces (−)‐**1**,^[33]^ reflecting the observation that terpenes and cationic intermediates towards them from plants and bacteria often represent different enantiomers.[^34^, ^35^, ^36^, ^37^] The coinciding absolute configuration of (−)‐**1** from *E. mammosa* may point to a biosynthesis by symbiotic bacteria in the gorgonian.^[38]^

### 2.2 Chemistry of germacrene A

**Scheme 4:**
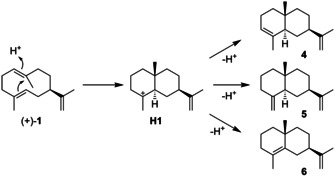
Acid catalysed conversion of **1** into selinenes.

The isolation and full structural and NMR‐spectroscopic characterisation of **1** was a long‐standing problem significantly hampered by its high reactivity. Its first isolation from *E. mammosa* in 1970 was done by extraction and concentration at temperatures below 35 °C to avoid the Cope rearrangement to **2** (Scheme 2).^[7]^ Chromatographic purification on slightly acidic silica gel induces a cyclisation through cation **H1** to α‐selinene (**4**), β‐selinene (**5**), and selina‐4,11‐diene (**6**, Scheme 4).[^7^, ^11^, ^15^]

**Scheme 5:**
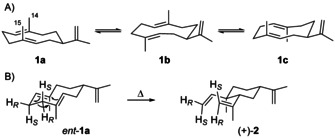
A) Conformers of (+)‐**1**. B) Cope rearrangement of *ent*‐**1 a**.

The skeleton of **1** is characterised by a conformationally flexible 10‐membered ring that shows sufficient ring strain to prevent a fast interconversion between conformers, resulting in broadened signals and multiple signal sets in the NMR spectra. Partial ^1^H‐ and ^13^C‐NMR data were first published for **1** from *T. maculata*.^[12]^ Later studies improved the NMR data assignments for the main conformers of **1** (recorded at 25 °C), but did not allow for a completion of the data sets.[^13^, ^39^] Through NOESY the conformers of **1 a** (UU, Me14 and Me15 up), **1 b** (UD, up‐down) and **1 c** (DU, down‐up) in a 5:3:2 ratio were identified (Scheme 5 A).^[13]^ The NMR data sets (25 °C) for all three conformers were recently completed using a ^13^C‐labelling strategy by conversion of all 15 isotopomers of (^13^C)FPP^[40]^ with GAS from *M. marina* into (−)‐**1**, resulting in strongly enhanced ^13^C‐NMR signals for the labelled carbons. HSQC spectroscopy of enzymatically prepared stereoselectively deuterated and ^13^C‐labelled **1** allowed the NMR assignment of all hydrogens.[^33^, ^41^]

The stereoselectively deuterated and ^13^C‐labelled isotopomers of **1** were also used to study the stereochemical course of its Cope rearrangement (Scheme 5 B). According to the Woodward–Hoffmann rules, pericyclic reactions follow a stereochemical course determined by the symmetry of frontier orbitals.^[42]^ For the Diels–Alder reaction this has been verified by stereoselective deuteration,[^43^, ^44^] while classical experiments for the Cope rearrangement have been performed with *meso*‐ and *rac*‐3,4‐dimethylhexa‐1,5‐diene.^[45]^ The enzymatic access to labelled **1** allowed to follow the rearrangement to (+)‐**2** that proceeds from *ent*‐**1 a** through a chair‐chair transition state.^[33]^

**Scheme 6:**
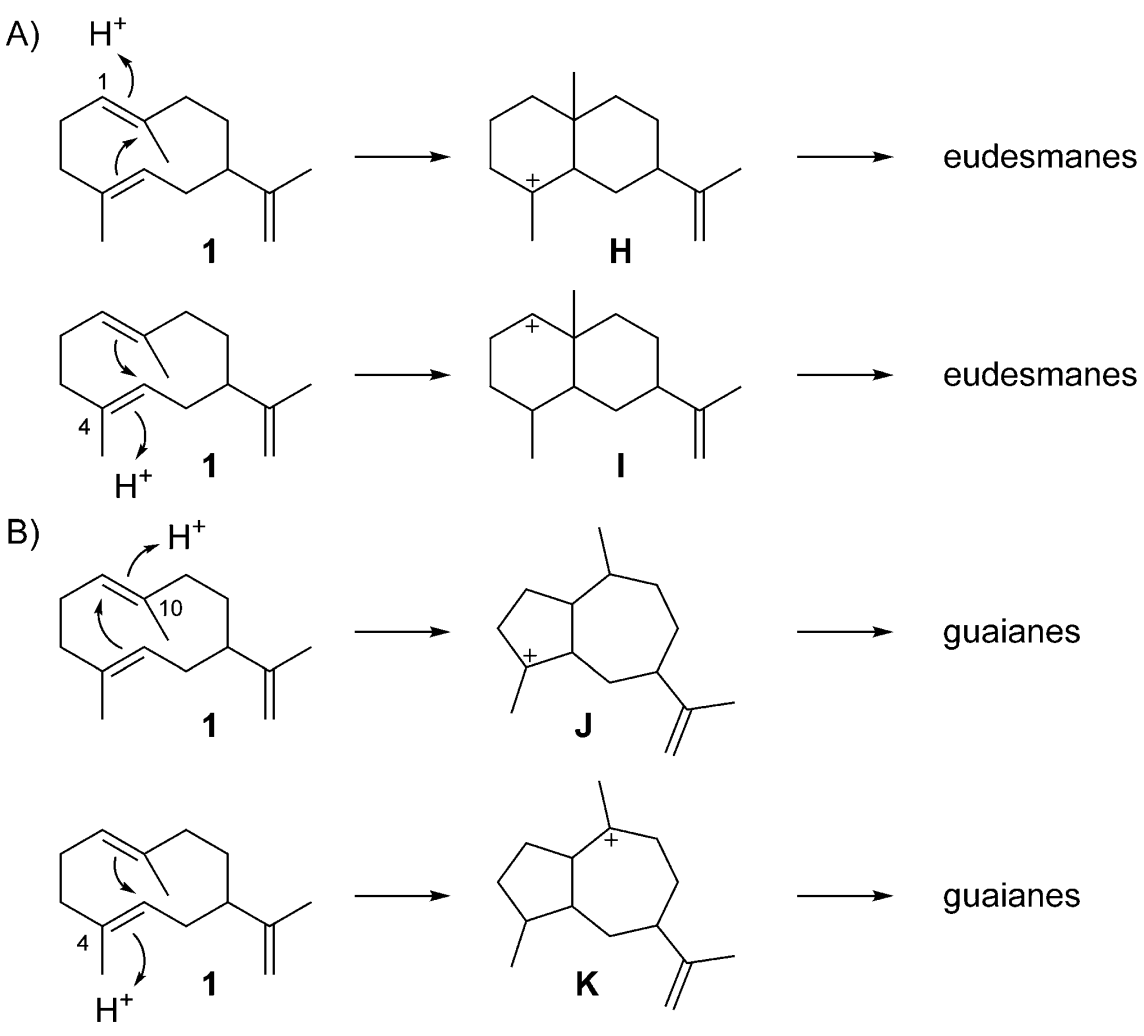
Secondary terpene cyclisations of **1**.

For many terpene synthase reactions **1** is further cyclised in a second step initiated by reprotonation. This can occur at C1 and lead to the 6–6 bicyclic system of **H** as a precursor of eudesmane sesquiterpenes (Scheme 6 A). The 6–6 bicyclic system could in theory also arise by protonation at C4 leading to the secondary cation **I**, but this reaction is not preferred. Furthermore, **1** can be protonated at C10 with cyclisation to the 5–7 bicyclic skeleton of **J**, or at C4 resulting in **K**, representing the precursors to guaiane sesquiterpenes. As an alternative to the formation of neutral **1** and its reportonation also an intramolecular or water‐mediated proton transfer in cation **B** may directly lead to **H**, **J** or **K**, thus bypassing **1** that would in such cases be better described as a side product rather than an intermediate. However, experimental evidence to distinguish between these alternatives is difficult to obtain, and **1** will preferentially be discussed as an intermediate towards more complex sesquiterpenes in this article. A detailed discussion of the reactions from **1** will follow in the subsequent sections.

## 3 Eudesmanes

### 3.1 Eudesmanes with a regular skeleton

**Scheme 7:**
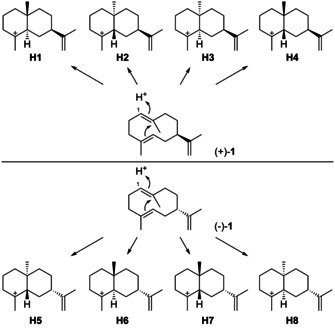
Cyclisations induced by reprotonation of **1** at C1 to **H1**–**H8**.

The protonation‐induced cyclisation of **1** can lead to eight stereochemically distinct cationic intermediates (Scheme 7), four of which arise from (+)‐**1** (**H1**–**H4**), while the other four stereoisomers originate from (−)‐**1** (**H5**–**H8**). For each intermediate, simple deprotonations or nucleophilic attack of water are possible. Also, hydride shifts can occur first, which further widens the reachable chemical space of eudesmanes. For many of these possibilities the corresponding structures have been reported.

### 3.2 Eudesmanes from cation H1

**Scheme 8:**
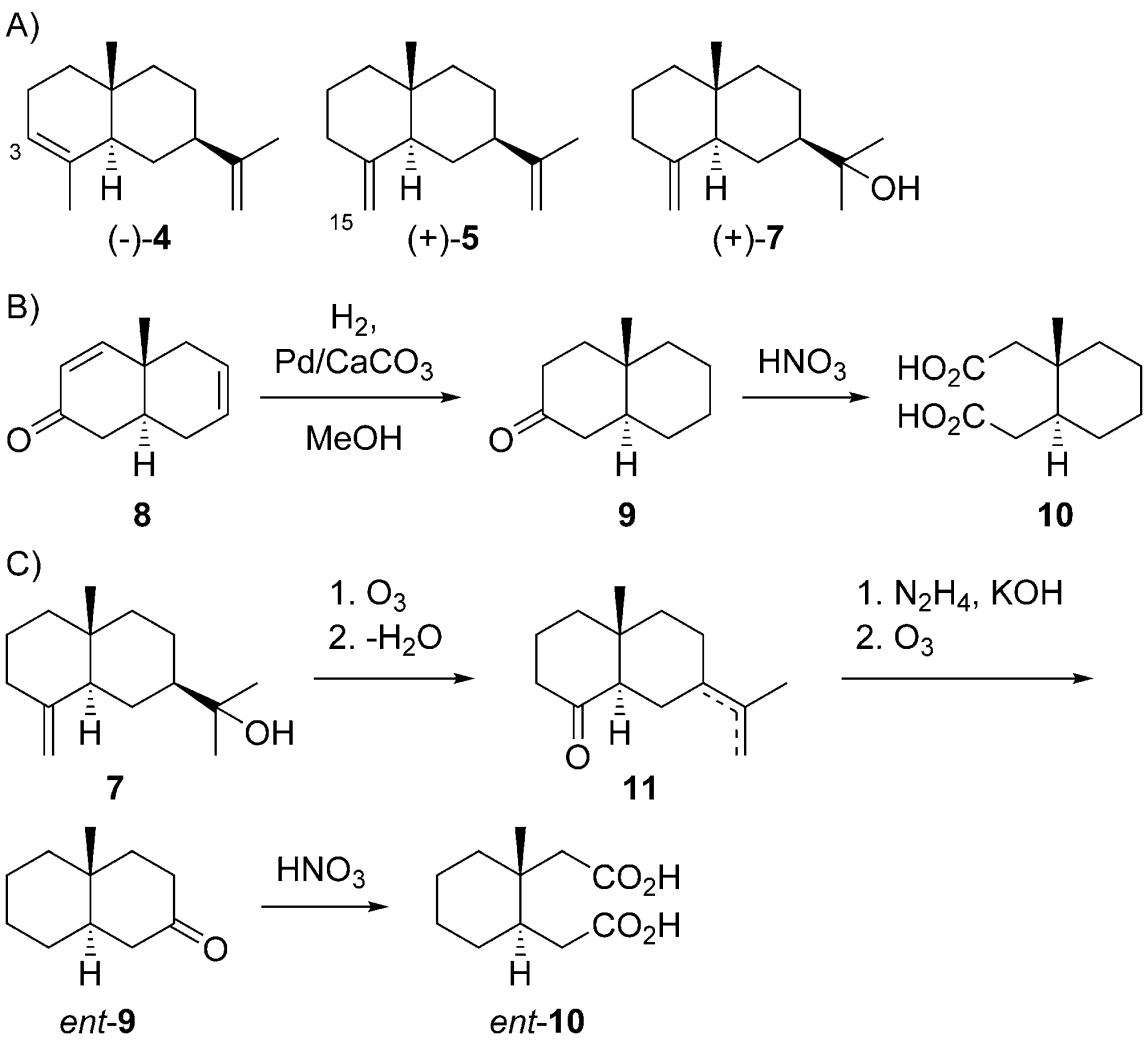
A) Structures of eudesmanes from **H1** and of **7**. B) Chemical correlation of ketone **8** with **10**. C) Chemical correlation of **7** with *ent*‐**10**.

An important intermediate to eudesmanes is **H1**. Deprotonations from C3 and C15 lead to α‐selinene (**4**) and β‐selinene (**5**), two compounds that have been isolated more than 100 years ago from celery oil.^[46]^ Their structures were elucidated in degradation experiments^[47]^ and were correlated to β‐eudesmol (**7**, Scheme 8 A).[^48^, ^49^, ^50^] Based on a comparison of physical characteristics of degradation products to those of other *cis*‐ and *trans*‐decalins initially a *cis*‐decalin structure was assigned,^[51]^ but a later conformational re‐examination indicated a *trans*‐fused ring system.[^52^, ^53^] The absolute configurations of **4** and **5** were determined by chemical correlation through the following arguments. The structure of ketone **8** was established in the classical synthesis of steroids by Woodward.^[54]^ Two years later the same group converted **8** into the dicarboxylic acid **10** (Scheme 8 B) that was the opposite enantiomer as obtained by degradation of **7** (Schemes 8 C)^[55]^ that had previously been correlated with **4** and **5** (vide supra).

The optical rotation of **4** was repeatedly found to have a positive value, including the reports from Brazilian rosewood oil ([α]_D_=+18),^[56]^ *Dendropanax trifidus* ([α]_D_=+68)^[57]^ and *Cryptotaenia japonica* ([a]_D_^15^=+6.3),^[58]^ or for **4** obtained by enantioselective synthesis ([α]_D_=+15.7, CHCl_3_).^[59]^ Andersen et al. pointed out that minor impurities may result in erroneous data and reported a value of [α]_D_=−16 (*c* 0.2, pentane)^[60]^ that was confirmed by Maurer and Grieder ([α]_D_^20^=−14.5, CHCl_3_, 1 %),^[61]^ and in both cases secured by CD spectroscopy. For **5** consistently positive optical rotations with values between [α]_D_=+31.7 (CHCl_3_) and [α]_D_=+60 (CHCl_3_) have been given.[^49^, ^57^, ^58^, ^60^, ^61^, ^62^, ^63^, ^64^, ^65^, ^66^] Thus, natural α‐ and β‐selinene from (+)‐**1** are characterised as (−)‐**4** and (+)‐**5**. Complete ^1^H‐ and ^13^C‐NMR data for **4** and **5** are available.[^66^, ^67^]

Compounds **4** and **5** were identified from various plant sources.[^49^, ^57^, ^58^, ^60^, ^61^, ^62^, ^63^, ^66^, ^67^, ^68^, ^69^, ^70^, ^71^, ^72^, ^73^, ^74^, ^75^, ^76^] In some cases **2** was also isolated,[^58^, ^68^, ^69^] sometimes with determined absolute configuration of (−)‐**2**,[^61^, ^62^, ^63^] which supports (+)‐**1** as a biosynthetic intermediate, but **1** could also be the true natural product, while **4** and **5** may have been formed spontaneously from **1** during compound isolation (Scheme 4).

**Scheme 9:**
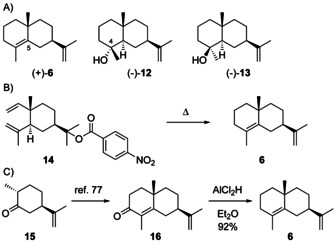
A) Structures of eudesmanes from **H1**. B) Correlation of **14** to **6**. C) Synthesis of **6**.

An alternative deprotonation of **H1** can lead to selina‐4,11‐diene (**6**), while the attack of water may result in selin‐11‐en‐4α‐ol (**12**) or neointermedeol (**13**, Scheme 9 A). As the stereochemical information at C5 is lost in **6**, this sesquiterpene can also arise from **H4**. Conclusions may be possible from co‐isolated materials with retained stereochemical information at C5. The absolute configuration of **6** was evident from its formation by pyrolysis of the *p*‐nitrobenzoate **14** of (−)‐elemol (**3**), leading to (+)‐**6** (Scheme 9 B).^[65]^ This finding is further supported by an enantioselective synthesis of (+)‐**6** starting from (+)‐*trans*‐dihydrocarvone (**15**) through (+)‐α‐cyperone (**16**),^[77]^ followed by reduction of the ketone with AlCl_2_H (Scheme 9 C).^[78]^

Compound **6** has been isolated from several plants[^62^, ^74^, ^79^, ^80^, ^81^, ^82^, ^83^] with reported positive optical rotations ranging from [α]_D_^14^=+32.05 (MeOH)^[79]^ to [α]_D_^20^=+54.5 (CHCl_3_, 1 %).^[80]^ From *Vernonia glabra* **6** was isolated together with **1**, **2**, **4** and **5** after column chromatography, suggesting that it may have been formed by silicic‐acid‐catalysed cyclisation of **1**.^[81]^ The full^[61]^ or partial[^78^, ^79^, ^84^] ^1^H‐NMR data have frequently been published, but unfortunately no ^13^C‐NMR data are available from the literature.

**Scheme 10:**
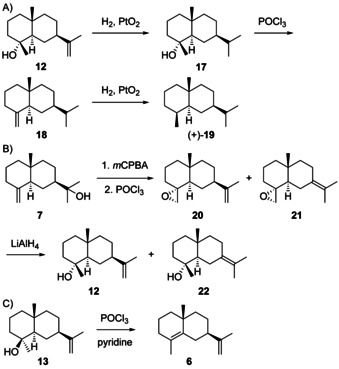
Chemical correlations of **12** with A) **19** and B) **7**. C) Correlation of **13** with **6**.

The alcohol **12** ([α]_D_^20^=−18) was first isolated from *Podocarpus dacrydioides* and its structure was correlated to (+)‐selinane (**19**), the hydrocarbon corresponding to **4** and **5**, by catalytic hydrogenation to **17**, dehydration with POCl_3_ to **18** and hydrogenation (Scheme 10 A), while the 4α orientation of the hydroxy function was deduced from the NMR spectrum, thereby establishing its absolute configuration.^[85]^ This structural assignment was confirmed by a synthesis from **7** that was converted into the epoxide and dehydrated with POCl_3_ to yield a mixture of **20** and **21** (Scheme 10 B). Epoxide opening with LiAlH_4_ resulted in (−)‐**12** and juniper camphor (**22**).^[86]^ Furthermore, the racemic compound, along with all other seven stereoisomers, has been synthesised^[87]^ and comparative spectroscopic data including ^1^H‐ and ^13^C‐NMR have been published.[^87^, ^88^] Identical ^1^H‐ and ^13^C‐NMR data for **12** were reported for the material from *Artemisia barrelieri* ^[89]^ and *Tanacetum nubigenum*.^[90]^ Compound **12** has been isolated from many plant species.[^73^, ^80^, ^82^, ^89^, ^90^, ^91^, ^92^, ^93^, ^94^, ^95^, ^96^, ^97^, ^98^, ^99^, ^100^, ^101^, ^102^]

Neointermedeol (**13**) was first reported from the grass *Bothriochloa intermedia*, with an optical rotation of [α]_D_^25^= +7.5,[^103^, ^104^] while the material isolated later from *Panax ginseng* exhibited a negative optical rotation ([α]_D_^22^=−4.8, *c* 3.45, CHCl_3_).^[84]^ To resolve the situation (−)‐**13** was dehydrated with POCl_3_ in pyridine, yielding (+)‐**6** and thus securing the absolute configuration of **13** (Scheme 10 C). The structure of **13** has also been confirmed by synthesis of the racemate.^[87]^ Further isolations have been reported from termites including *Subulitermes baileyi* ^[105]^ and *Amitermes excellens*,^[106]^ and from the plants *Geigeria burkei* ^[107]^ and *Artemisia schmidtiana*.^[108]^ Partial ^1^H‐ and full ^13^C‐NMR data for **13** have been published.[^84^, ^104^]

### 3.3 Eudesmanes from cation H2

**Scheme 11:**
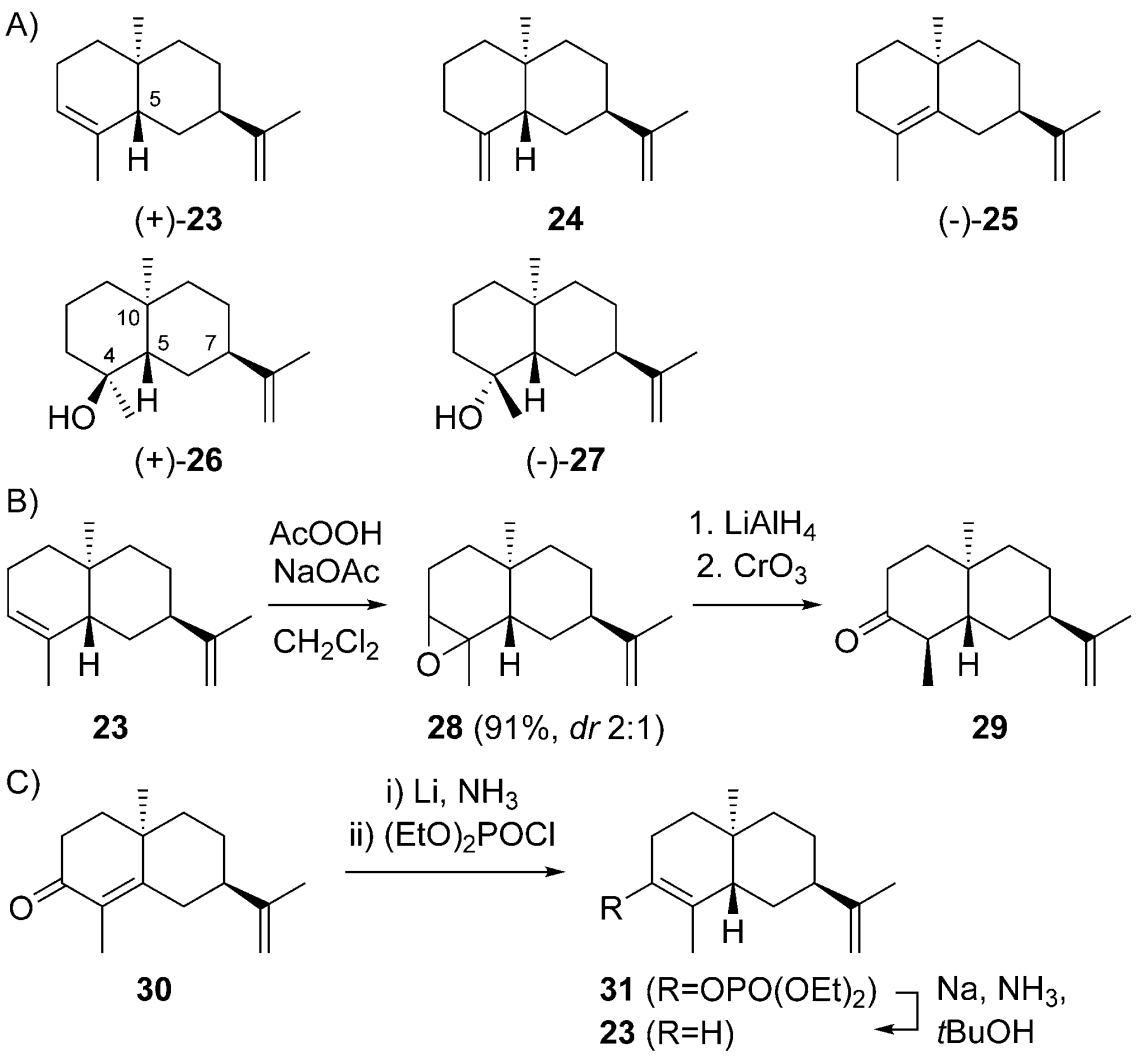
A) Structures of **23**–**26** and “paradisiol” (**27**). Chemical correlations of **23** with B) ketone **29** and C) synthetic **30**.

Sesquiterpenes arising through **H2** occur less frequent in Nature compared to **H1** derivatives, but the alcohol **26** (Scheme 11 A) is quite widespread. The sesquiterpene 5,10‐*diepi*‐α‐selinene (**23**) was first reported from *Dipterocarpus alatus* ([α]_D_^20^=+2.1).^[109]^ The compound was co‐isolated with (7*R*,10*S*)‐eudesma‐4,11‐diene, (−)‐**25** ([α]_D_^20^=−108.6), that could potentially also arise by deprotonation of **H3**, but if a common terpene cyclisation is assumed, intermediate **H2** should be relevant. The absolute configuration of **23** was assigned by epoxidation with peracetic acid to a mixture of stereoisomeric epoxides **28**, reduction with LiAlH_4_ to yield a mixture of alcohols, and Jones oxidation. From the obtained ketones **29**, the enantiomer of a known compound, was isolated as main product (Scheme 11 B).^[109]^ Further, an enantioselective synthesis of **23** from **30** that is readily accessible from dihydrocarvone **15** was reported, that proceeded by reduction with Li in NH_3_ and phosphorylation with (EtO)_2_POCl to **31**, followed by defunctionalisation with Na in NH_3_ and *t*BuOH (Scheme 11 C).^[110]^ Alternatively, **30** can be converted into a mixture of **23**, its C5 epimer and **25** by Wolff–Kishner reduction.^[111]^ The regioisomer 5,10‐*diepi*‐β‐selinene (**24**) was first obtained along with **23** by dehydration of a sesquiterpene alcohol with the assigned structure of “paradisiol” (**27**) from grapefruit (*Citrus paradisi*).^[112]^ Subsequent work demonstrated that “paradisiol” was identical with intermedeol (**26**).^[113]^ All three compounds **23**–**25** were also obtained by hydrolysis of intermedeol β‐d‐fucopyranoside.^[114]^ Compound **23**, sometimes accompanied by **24** or **25**, has also been reported from several termites.[^106^, ^115^, ^116^] Full ^1^H‐ and ^13^C‐NMR data of **23** (with missing signals only for quaternary olefinic carbons) and **24** are available from the literature,^[116]^ while data for **25** are lacking.

**Scheme 12:**
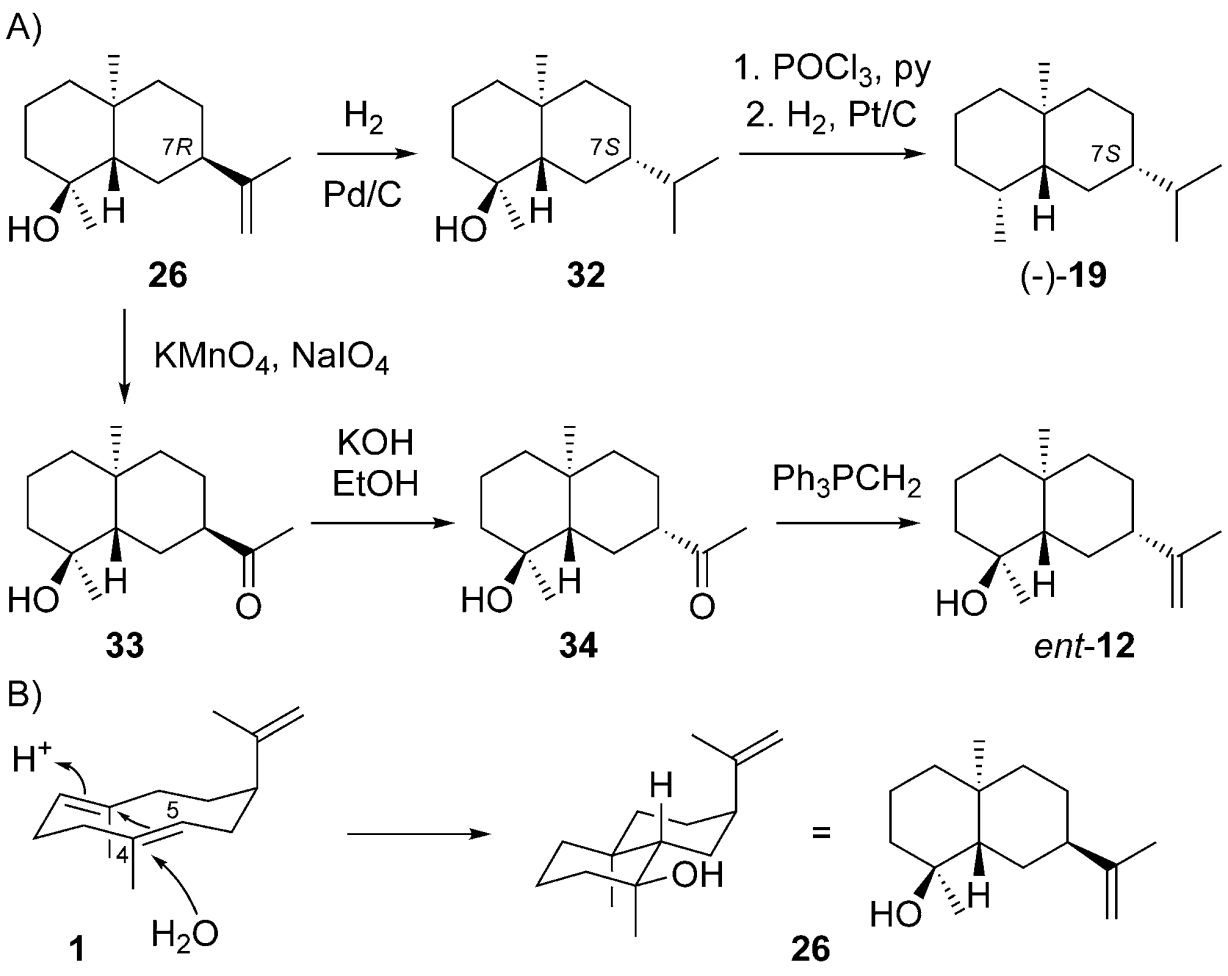
A) Chemical correlations of **26** with (−)‐**19** and *ent*‐**12**, B) concerted mechanism for the protonation induced cyclisation of **1** to **26**.

Intermedeol (4*S*,5*S*,7*R*,10*S*)‐**26** ([α]_D_^25^=+10.7) was first reported with 7*S* configuration from *Bothriochloa intermedia*.^[117]^ This wrong structural assignment was based on the finding that **26** was converted into (−)‐selinane (**19**) by hydrogenation (Pd/C), dehydration (POCl_3_, pyridine) and hydrogenation (Pt/C, Scheme 12 A). The subsequently discovered alcohol **12** (Scheme 9)^[85]^ showed different physical characteristics and spectroscopic properties, and thus the structure of *ent*‐**12** for intermedeol was excluded. Oxidation of **26** with KMnO_4_ and NaIO_4_ to hydroxyketone **33**, followed by epimerisation to **34** and Wittig methylenation gave *ent*‐**12**, supporting a structural revision for intermedeol to **26**. The initially observed formation of (−)‐**19** from **26** was explained by double bond migration and hydrogenation from the sterically less hindered side during Pd catalysis, yielding intermediate **32** with overall epimerisation at C7.^[86]^ The structure of **26** was also confirmed by synthesis.[^87^, ^110^, ^111^]

Compound **26** has frequently been isolated from plants.[^66^, ^117^, ^118^, ^119^, ^120^, ^121^, ^122^, ^123^, ^124^, ^125^, ^126^, ^127^] For **26** isolated from *Cymbopogon flexuosus* the opposite absolute configuration was assigned, despite the optical activity of [α]_D_=+2 (*c* 3.3, MeOH). The compound was named “isointermedeol”,^[128]^ but this material was likely an impure sample of (+)‐**26**.^[129]^ Nevertheless, the description of “isointermedeol” caused some confusion, as there is at least one later paper about *Jasonia candicans* with reference to the report of this supposedly new sesquiterpene alcohol.^[130]^ For the (+)‐intermedeol synthase from *Termitomyces* GC‐MS analysis of the products revealed minor amounts of **2**, thereby establishing **1** as a side product and supporting this compound as a biosynthetic intermediate to **26**.^[131]^ Another (+)‐intermedeol synthase was recently reported from *Streptomyces clavuligerus*.^[132]^ Complete ^1^H‐ and ^13^C‐NMR data of **26** in CDCl_3_[^87^, ^88^, ^104^, ^114^, ^119^] or C_6_D_6_[^131^, ^132^] have been reported.

Paradisiol (4*R*,5*S*,7*R*,10*S*)‐**27** represents the initially assigned structure of a sesquiterpene alcohol from *Citrus paradisi* ^[112]^ that was later corrected to **26**.^[113]^ It may seem surprising that **27** has never been reported as a natural product, while its epimer **26** is widespread, but this is understandable on biosynthetic grounds (Scheme 12 B). Starting from the shown conformation of **1**, a concerted protonation induced ring closure and attack of water can lead to **26**, while the formation of **27** by such a process would require a *syn* addition to the C4=C5 double bond of **1** with attack of water from the internal face, which seems sterically impossible. However, compound **27** has been synthesised^[87]^ and was obtained as one of the hydrolysis products of intermedeol β‐d‐fucopyranoside ([α]_D_^22^=−17.9, *c* 0.53, EtOH).^[114]^ Full spectroscopic data are available.[^87^, ^88^, ^114^]

### 3.4 Eudesmanes from cation H3

**Scheme 13:**
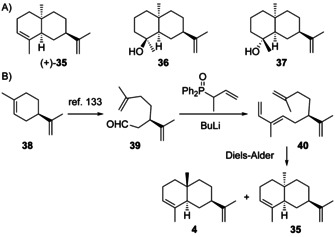
A) Structures of **35**–**37**. B) Enantioselective synthesis of **35**.

Natural products from **H3** are unknown. Synthetic compounds that could formally arise through **H3** by terpene cyclisation include 10‐*epi*‐α‐selinene (**35**), 7‐*epi*‐amiteol (**36**) and 5‐*epi*‐paradisiol (**37**, Scheme 13). Compound **35** was first obtained by Wolff–Kishner reduction of **30**,^[111]^ and then from (*R*)‐limonene (**37**) that can be converted in three steps into the aldehyde **39** (Scheme 13 B),^[133]^ followed by Wittig–Horner olefination to **40**. An intramolecular Diels–Alder reaction results in the *endo*‐adduct **4** and the *exo*‐adduct **35** ([α]_D_^25^=+102, CHCl_3_, 0.7 %).^[134]^ A similar route was also reported from (*S*)‐carvone.^[135]^ For **36** and **37** only synthetic routes to the racemates have been established.^[87]^ For all three compounds full spectroscopic data have been published.[^87^, ^88^, ^135^]

### 3.5 Eudesmanes from cation H4

**Scheme 14:**
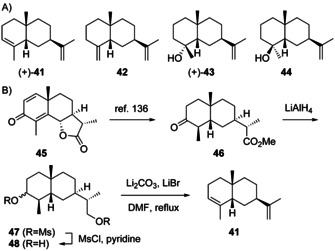
A) Structures of **41**–**44**. B) Enantioselective synthesis of **41**.

Only a few natural products arising through **H4** are known. Amiteol (+)‐**43** ([α]_365_^24^=+8, CHCl_3_) from the termite *Amitermes excellens* was the first isolated compound from this class and co‐occurred with 5‐*epi*‐α‐selinene (**41**), 5‐*epi*‐β‐selinene (**42**) and **6** in this species (Scheme 14 A).^[107]^ Although **6** is usually assumed to be formed via **H1**, in *A. excellens* a formation via **H4** is more likely, as this reflects the mechanism for its cometabolites. The absolute configuration of **43** was established by dehydration with SOCl_2_, yielding a mixture of **41**, **42** and (+)‐**6** ([α]_D_^24^=+30, CHCl_3_),^[106]^ the same enantiomer as originally reported from *Chamaecyparis formosensis*.^[79]^ Furthermore, (+)‐**41** was synthesised from α‐santonin (**45**) that was converted into **46** through a known route (Scheme 14 B).^[136]^ Reduction of **46** to epimeric diols **47**, mesylation to **48** and elimination with Li_2_CO_3_ and LiBr in refluxing DMF yielded **41** ([α]_D_^25^=+30.1, *c* 3.50, CHCl_3_).^[137]^ Syntheses for racemic **43** and 5‐*epi*‐neointermedeol (**44**) have also been established,^[87]^ but despite its tentative GC/MS based identification as constituent of some essential oils compound **44** has not been isolated from natural sources so far. More recently, a terpene synthase for **41** has been identified from the cyanobacterium *Nostoc punctiforme*, but the absolute configuration of the product has not been assigned.^[138]^ Full spectroscopic data including IR, ^1^H‐ and ^13^C‐NMR are available for **41**,[^137^, ^138^] **43** and **44**.[^87^, ^88^]

**Scheme 15:**
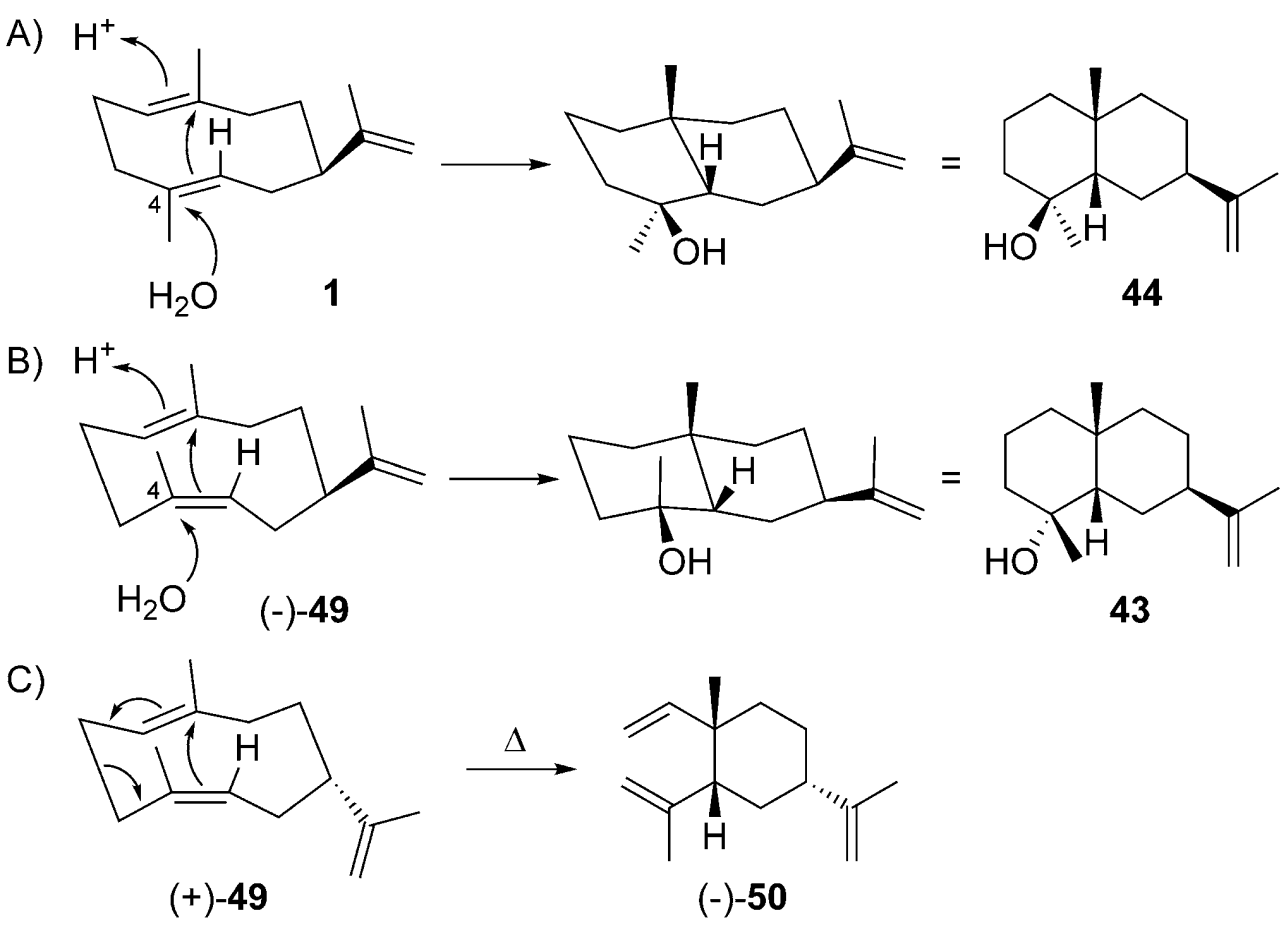
Protonation induced cyclisations A) of **1** to **44** and B) of **49** to **43**. C) Cope rearrangement of **49** to **50**.

Notably, while the formation of the sesquiterpene hydrocarbons **41**, **42** and **6** should be possible through **H4**, the formation of **43** along this pathway encounters a difficulty that is related to the explanation for the possible formation of **26**, but not of **27**, from **H2** (Scheme 12). Along similar lines (Scheme 15 A), the protonation induced cyclisation of **1** starting from a boat–boat conformation can explain the biosynthesis of **44**, while the formation of **43** would require the nucleophilic attack of water from the sterically less accessible *Re* face at C4. However, the formation of **43** is well understandable, if a precursor with a C4=C5 *Z*‐configured double bond would be assumed (Scheme 15 B). This precursor is known as (−)‐helminthogermacrene (**49**) from the fungus *Helminthosporium sativum* ^[139]^ and later from the termite *Amitermes wheeleri*.^[140]^ The enantiomer (+)‐**49** was reported from the liverwort *Scapania undulata* and has a very similar EI mass spectrum and GC retention index to **1**, but is less prone to a Cope rearrangement to (−)‐*cis*‐β‐elemene (**50**, Scheme 15 C).^[141]^ Synthetic routes towards racemic **49** have been developed[^139^, ^142^] and the absolute configuration of (+)‐**49** was established by chemical correlation to (−)‐helmiscapene, a compound discussed in Section 3.8.^[39]^

### 3.6 Eudesmanes from cation H5

**Figure 1:**
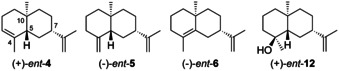
Structures of *ent*‐**4**–*ent*‐**6** and *ent*‐**12**.

Compounds derived from (−)‐**1** through the enantiomeric series of intermediates **H5**—**H8** have been reported less often compared to those from (+)‐**1**, which may be attributed to the fact that still most work has been done on higher plants for which (+)‐**1** is the typical enantiomer (Section 2). The cation **H5** gives rise to the known natural products *ent*‐α‐selinene (*ent*‐**4**), *ent*‐β‐selinene (*ent*‐**5**), *ent*‐selina‐4,11‐diene (*ent*‐**6**) and (4*S*,5*S*,7*S*,10*S*)‐eudes‐11‐en‐4‐ol (*ent*‐**12**, Figure 1).

The first report about naturally occurring enantiomers of selinane sesquiterpenes identified *ent*‐**4** as a constituent of the liverwort *Chiloscyphus polyanthus* in 1973. Its absolute configuration was established by CD spectroscopy in comparison to authentic (−)‐**4**.^[60]^ Compounds *ent*‐**4** and *ent*‐**6**, likewise established by CD spectroscopy and accompanied by **2**, were subsequently reported from the liverworts *Diplophyllum albicans* and *D. taxifolium*,^[143]^ while the liverworts *Riccardia jackii*, *Bazzania spiralis* and *Tylimanthus tenellus* contain different combinations of *ent*‐**4**, *ent*‐**5** and *ent*‐**12**.[^144^, ^145^, ^146^, ^147^] Also insects were reported to contain *ent*‐**4** and (+)‐**2**, exemplified by their occurrence in *Ceroplastes ceriferus*, which is surprising considering the fact that the „normal“ enantiomeric series of compounds is present in the related species *C. rubens*.^[62]^ In all these examples the absolute configurations were determined from the optical rotations of the isolated compounds. In *Penicillium roqueforti* also *ent*‐**4**, *ent*‐**5** and *ent*‐**12** may occur; in this case the absolute configurations were assigned based on their biosynthetic relationship to aristolochene (vide infra) that is generated through (−)‐**1** in this fungus.^[148]^

### 3.7 Eudesmanes from cation H6

**Scheme 16:**
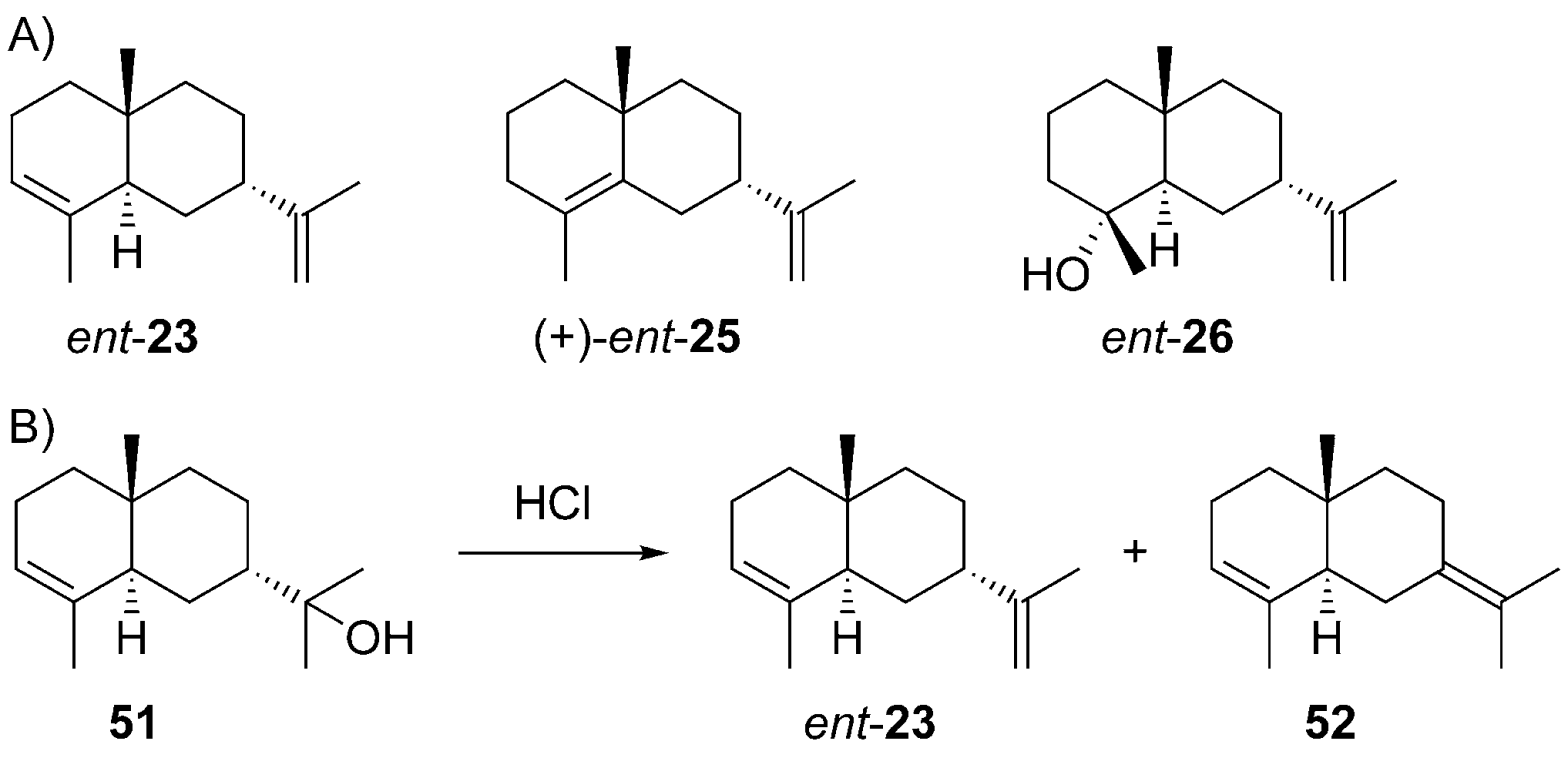
A) Structures of *ent*‐**23**, *ent*‐**25** and *ent*‐**26**. B) Dehydration of **51**.

Little is known about eudesmanes arising via cationic intermediate **H6**. The compound 7‐*epi*‐α‐selinene (*ent*‐**23**, Scheme 16 A) was first reported from *Amyris balsamifera*, a species from which also 7‐*epi*‐α‐eudesmol (**51**, Scheme 16 B) was isolated and structurally characterised by NMR spectroscopy. From its positive optical rotation ([α]_D_=+10, *c* 1.8, CHCl_3_) the authors concluded on the shown absolute configuration for **51**, but a comprehensible explanation for this assignment is missing. Dehydration of **51** yielded a mixture of two products to which the structures of *ent*‐**23** and **52** were assigned by NMR spectroscopy, unfortunately without separating the obtained materials and determining their optical rotations. The compounds described as *ent*‐**23** and **52** also occurred in the essential oil of *A. balsamifera*.^[149]^ One study reported the chromatographic separation of the compound from *A. balsamifera* and (+)‐**23** (the latter with a mentioned source „provided by Dr. Wilfried König“) on a chiral stationary GC phase, which represents the only hint in the literature that the structure of *ent*‐**23** for the essential oil constituent may be correctly assigned.^[150]^ Compound *ent*‐**23** was also reported as major product of a terpene synthase from *Vitis vinifera*.[^150^, ^151^] Both enantiomers of **23** have been obtained by synthesis from the enantiomers of **15**, but optical rotary powers of the products were not measured.^[152]^ However, *ent*‐**23** may have a negative optical rotation, as for **23** from *Dipterocarpus alatus* a low value of [α]_D_^20^=+2.1 was determined.^[109]^ This would be consistent with a report by König in which *ent*‐**23** was published as the (−)‐enantiomer, albeit only based on separation by gas chromatography using a chiral stationary phase without isolation.^[153]^

Compound *ent*‐**25** ([α]_D_^16^=+46.5, *c* 0.85, CHCl_3_) has been synthesised using the same strategy as for **6** (Scheme 9 C),^[78]^ but has not been isolated from any organism. The only report about *ent*‐**26** from *Monactis macbridei* by Bohlmann and co‐workers^[154]^ gives a reference to the erroneous “isointermedeol”^[128]^ that was corrected shortly after.^[129]^ Unfortunately, Bohlmann's paper does not give an optical rotation for the isolated material so that it is difficult to judge, if the authors of this study were aware of the misassignment of “isointermedeol” at the time of their publication. Overall, this discussion shows that compounds from **H6** are not only rare, but if they occur in the literature, the assignments of absolute configurations remain unclear. Since the compounds originate in all cases from higher plants, they may truly be the usual enantiomers, that is, **23**, **25** and **26**.

### 3.8 Eudesmanes from cations H7 and H8

**Scheme 17:**
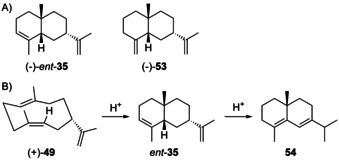
A) Structures of *ent*‐**35** and **53**. B) Acid‐catalysed cyclisation of **49** to *ent*‐**35** and isomerisation to **54**.

The literature contains only few reports of compounds that may originate from **H7**, while no examples from **H8** are available. α‐Helmiscapene (*ent*‐**35**, Scheme 17 A) was first isolated from *Scapania undulata* and suggested to arise through a “*cis*‐germacrene”,^[155]^ a compound that was later described from this species^[141]^ after its first identification from *H. sativum* as helminthogermacrene (**49**).^[139]^ In agreement with the positive optical rotation of synthetic **35** (Scheme 13), *ent*‐**35** was found to be the (−)‐enantiomer ([α]_D_=−100, CHCl_3_) and correlated to (+)‐δ‐selinene (**54**) by acid‐catalysed isomerisation (Scheme 17 B). Both *ent*‐**35** and β‐helmiscapene (−)‐**53** were also found in the liverwort *Radula perrottetii*.^[156]^ The acid‐catalysed cyclisation of (+)‐**49** to *ent*‐**35** suggests that the formation of *ent*‐**35** from **49** could be non‐enzymatic and that germacrene A may indeed not be the precursor of helmiscapenes.^[39]^ Full ^1^H‐ and ^13^C‐NMR data are available for *ent*‐**35** and **53**.[^39^, ^156^]

## 4 Rearranged Eudesmanes

In this section rearranged eudesmanes from **H1**–**H6** will be discussed, while such compounds from **H7** and **H8** are unknown.

### 4.1 Rearranged eudesmanes from H1

**Scheme 18:**
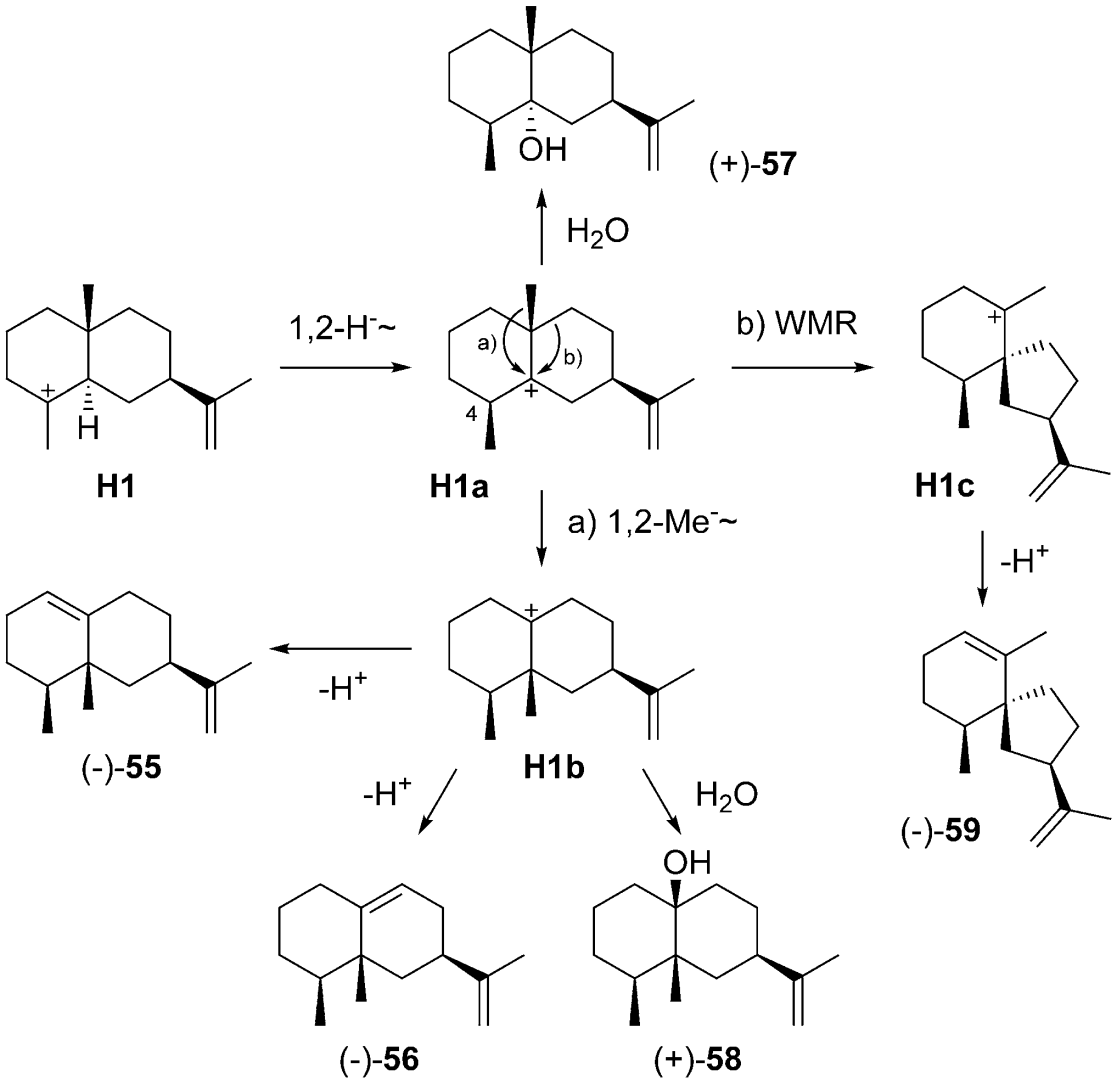
Biosynthesis of rearranged eudesmanes from **H1**.

Rearranged eudesmanes can in theory arise from all cations **H1**–**H8** in Scheme 7. An important group of compounds by widespread occurrence in Nature originates from **H1**. Specifically, this intermediate can undergo a 1,2‐hydride migration to **H1 a** that must proceed suprafacially and thus determines the configuration at C4 (Scheme 18; 1,n‐hydride or proton migrations as used in this article refer to the distance of n carbons for the migration, not to positional numbers). A subsequent 1,2‐methyl group migration leads to **H1 b** (path a) that upon deprotonation yields eremophilene (**55**) or 4,5‐*diepi*‐aristolochene (**56**). Alternatively, **H1 a** can react in a Wagner–Meerwein rearrangement (WMR) with ring contraction to **H1 c** that results in hinesene (**59**, path b).

Compound **55** was first isolated from *Petasites officinalis* and *P. albus* ([α]_D_^20^=−104.2 and [α]_D_^24^=−142.5, respectively).[^157^, ^158^, ^159^] Its structure was initially wrongly assigned,^[160]^ but then corrected based on a chemical derivatisation and interpretation of the EI‐MS fragmentation behaviour of a thioketal derivative.^[159]^ The sesquiterpene **55** was later isolated from several higher plants.[^58^, ^161^, ^162^, ^163^, ^164^, ^165^, ^166^, ^167^] Furthermore, (−)‐**55** was discovered in the gorgonian *Plexaurella fusifera* ^[168]^ and along with **2** in the liverwort *Frullania serratta*.^[169]^

**Scheme 19:**
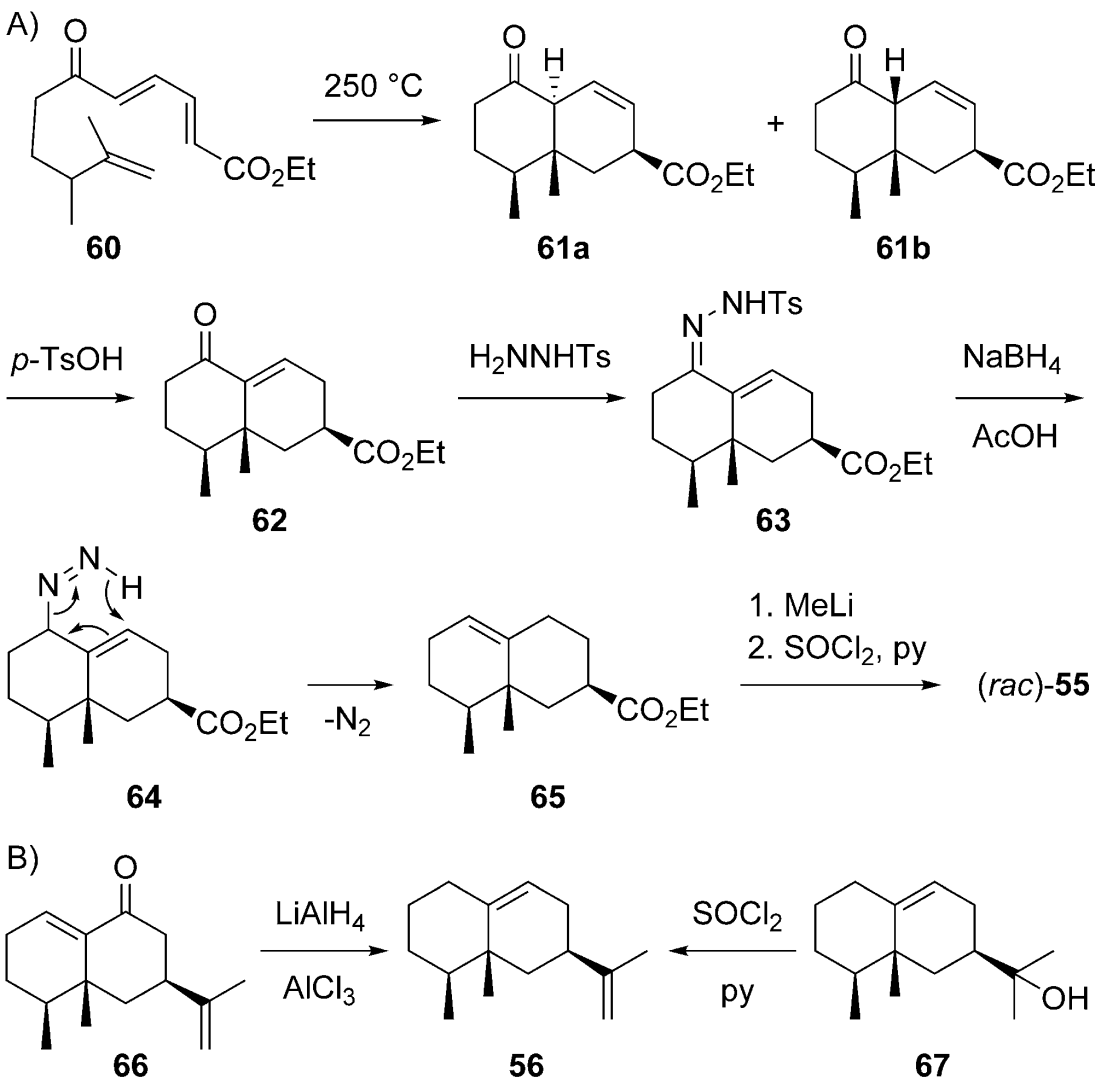
A) Synthesis of (*rac*)‐**55** through a Diels–Alder approach, B) preparation of **56** from the natural products **66** and **67**.

An elegant synthesis for (*rac*)‐**55** has been developed starting from **60** that can give **61 a** by a Diels–Alder reaction, with partial epimerisation to **61 b** (Scheme 19 A). Both compounds can be converted into **62** by acid‐catalysed isomerisation. Reaction with tosylhydrazine leads to **63** that was reduced with NaBH_4_ via **64** to **65**.^[170]^ Treatment with MeLi and dehydration with SOCl_2_ in pyridine gave **55**.^[171]^ Its double bond regioisomer **56** (Scheme 19 B) was first obtained from eremophilone (**66**), the first structurally characterised terpene found to violate Ruzicka's isoprene rule,^[172]^ by reduction with LiAlH_4_ and AlCl_3_,^[173]^ and later from eremophil‐9‐en‐11‐ol (**67**) by dehydration ([α]_D_=−11.1, *c* 0.18, CHCl_3_).^[174]^ Compound **56** has also been obtained by synthesis from capsidiol,^[175]^ but was never isolated from Nature. Complete ^13^C‐NMR data are available for **55** and **56**.[^170^, ^175^]

The sesquiterpene alcohol 4αH‐eudesma‐11‐en‐4α‐ol (**57**), [α]_D_=+32.8 (*c* 0.7, CHCl_3_), was isolated from *Kleinia pendula* and can arise by attack of water to **H1 a**.^[176]^ Similarly, the addition of water to **H1 b** leads to eremophil‐11‐en‐10β‐ol (**58**), a compound that is known from *Alpinia intermedia* ([α]_D_= +29.2, *c* 0.12, CHCl_3_).^[66]^ For both alcohols **57** and **58** full ^13^C‐NMR data were given.^[66]^

Hinesene (**59**) was first isolated from *Rolandra fruticosa* ([α]_D_^24^=−44, *c* 0.1, CHCl_3_).^[177]^ The absolute configuration was initially assigned based on the same sign of optical rotation than for hinesol and later confirmed by enantioselective synthesis from santonin.^[178]^ The compound is also known from an unspecified liverwort of the genus *Frullania*.^[179]^ Full ^1^H‐ and ^13^C‐NMR data were provided.[^177^, ^178^]

### 4.2 Rearranged eudesmanes from H2

**Scheme 20:**
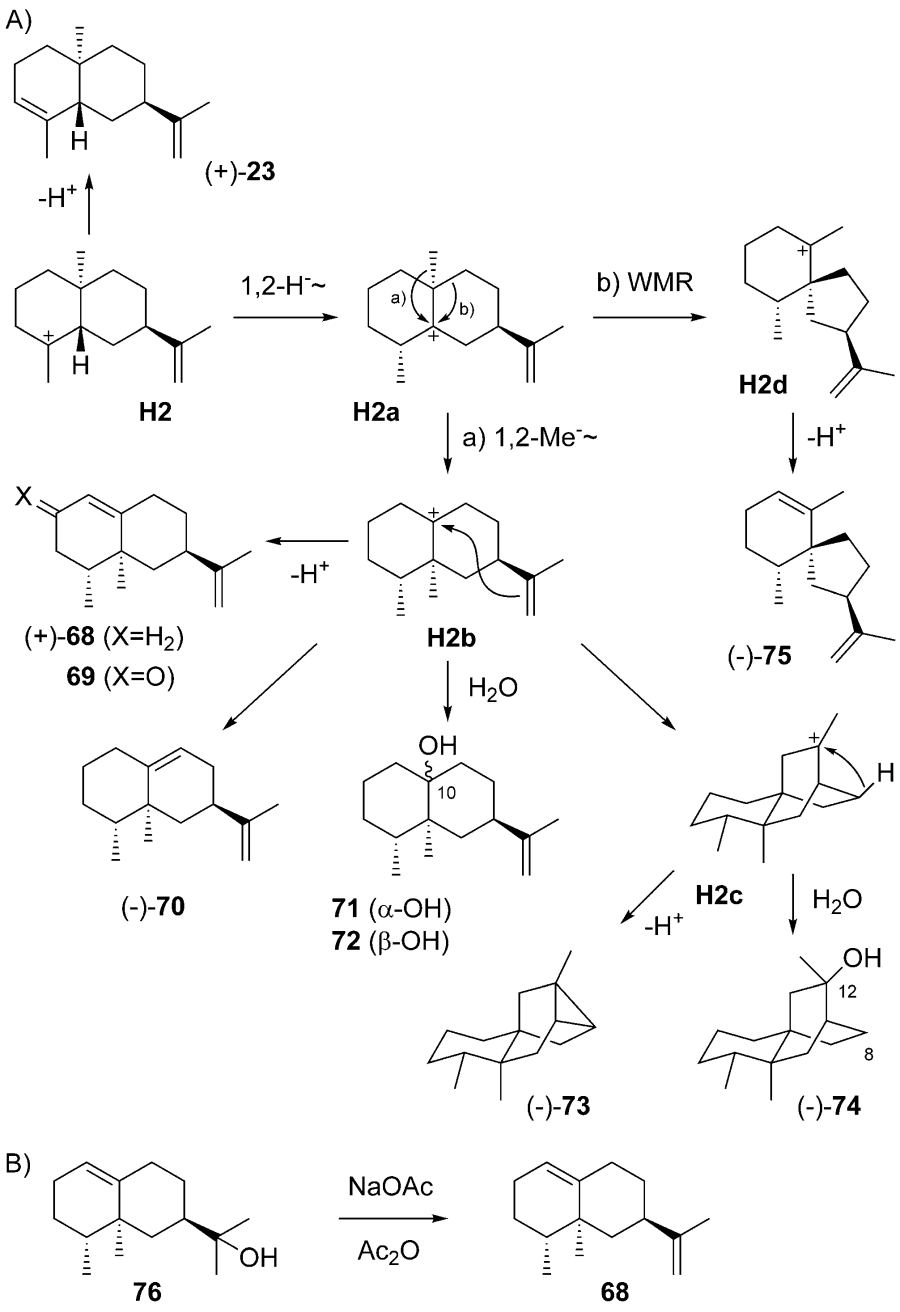
A) Biosynthesis of compounds from **H2**. B) Dehydration of **76** to **68**.

Also rearranged eudesmanes from **H2** constitute an important group of compounds (Scheme 20 A), including (+)‐valencene (**68**), (−)‐aristolochene (**70**), valencene hydrate (**71**) and its C10 epimer **72**, (−)‐ishwarane (**73**), (−)‐8,12‐*seco*‐ishwaran‐12‐ol (**74**) and (−)‐agarospirene (**71**). Compound **73** requires a third cyclisation from **H2 b** to **H2 c** and deprotonation with closure of a cyclopropane ring, while **74** can be explained by attack of water to **H2 c**.

Valencene (**68**) was first isolated from orange oil^[180]^ and found to be related to nootkatone (**69**) by oxidative conversion,^[181]^ an important value adding transformation for which an artificial enzyme system has been developed.^[182]^ Compound **69** is a flavour constituent of citrus fruits and its structure had previously been established.^[183]^ The optical rotation of **68** was determined for the material obtained by dehydration of valerianol (**76**, Scheme 20 B) with NaOAc in refluxing Ac_2_O ([α]_D_=+73.4, *c* 5.3, CHCl_3_).^[184]^ A synthesis of (*rac*)‐**68** similar to the synthesis of (*rac*)‐**55** in Scheme 19 A has been developed.^[170]^ The sesquiterpene **68** is a constituent of the essential oils from numerous plants, but has rarely been isolated. *Bixa orellana* is one of the few sources from which its isolation was mentioned,^[185]^ while it was obtained enriched together with **2** in a sesquiterpene hydrocarbon fraction from the liverwort *Porella acutifolia*.^[186]^ The combination of **2** and **68** also occurs in the octocoral *Plexaurella fusifera*,^[168]^ while **68** from bacteria is rare, but has been identified from *Streptomyces* sp. FORM5.^[187]^ Valencene synthases are known from *Citrus sinensis*,^[188]^ *Vitis vinifera*,[^150^, ^151^] and *Callitropsis nootkatensis*,^[189]^ in which it occurs together with a valencene oxidase for the biosynthesis of **69**.^[190]^ Besides **68**, the terpene synthases from *V. vinifera* were reported to produce (−)‐7‐*epi*‐selinene (*ent*‐**23**, Scheme 16)[^150^, ^151^] that must originate from **H6**. It would be easier to understand, if one of the two enzyme products would represent the opposite enantiomer than reported, so that both could arise through a common intermediate. In fact, the configurational assignment for **68** was based on a GC analysis using a chiral stationary phase, but without including a (−)‐**68** standard.

**Scheme 21:**
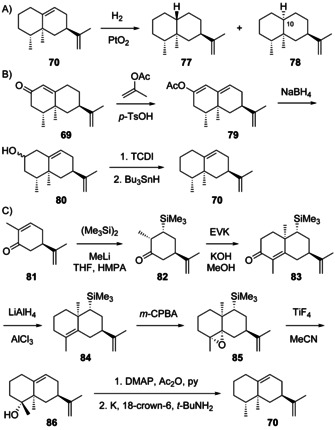
A) Hydrogenation of **70**. B) Synthesis of **70** from **69**, and C) from (*S*)‐carvone (**81**).

Aristolochene (**70**, [α]_D_^25^=−76.47) was first isolated from *Aristolochia indica*. Its structure was elucidated by NMR spectroscopy and catalytic hydrogenation, yielding a mixture of (+)‐nootkatane (**77**), also obtained by hydrogenation of **68**, and its C10 epimer **78** (Scheme 21 A).^[191]^ The structural assignment was later confirmed by a synthesis of **70** from **68**, that was first oxidised to **69**, followed by conversion into the dienol acetate **79** (Scheme 21 B). Deconjugation by reduction with NaBH_4_ gave **80** that was defunctionalised with thiocarbonyldiimidazole and Bu_3_SnH to yield **70**.^[192]^ Furthermore, an enantioselective synthesis from (*S*)‐carvone (**81**) has been developed (Scheme 21 C). After silylation to **82**, a Robinson annelation with ethylvinyl ketone resulted in **83**. Its reduction with excess LiAlH_4_ and AlCl_3_ to **84** was followed by epoxidation to **85**. Treatment with TiF_4_ resulted in epoxide opening with methyl group migration and cleavage of the trimethylsilyl cation to produce **86**, that was defunctionalised in two more steps to **70**.^[193]^ Compound **70** was also reported as a side product of valencene synthase from *V. vinifera* ^[150]^ and as a headspace constituent from *Streptomyces acidiscabies*.^[194]^ Both compounds (+)‐**68** and (−)‐**70** are present in extracts from the liverwort *Dumortiera hirsuta* with absolute configurations established in comparison to authentic standards by GC using a chiral stationary phase.^[153]^ Full ^13^C‐NMR data for **68** ^[170]^ and **70**[^193^, ^195^, ^196^] have been reported.

**Scheme 22:**
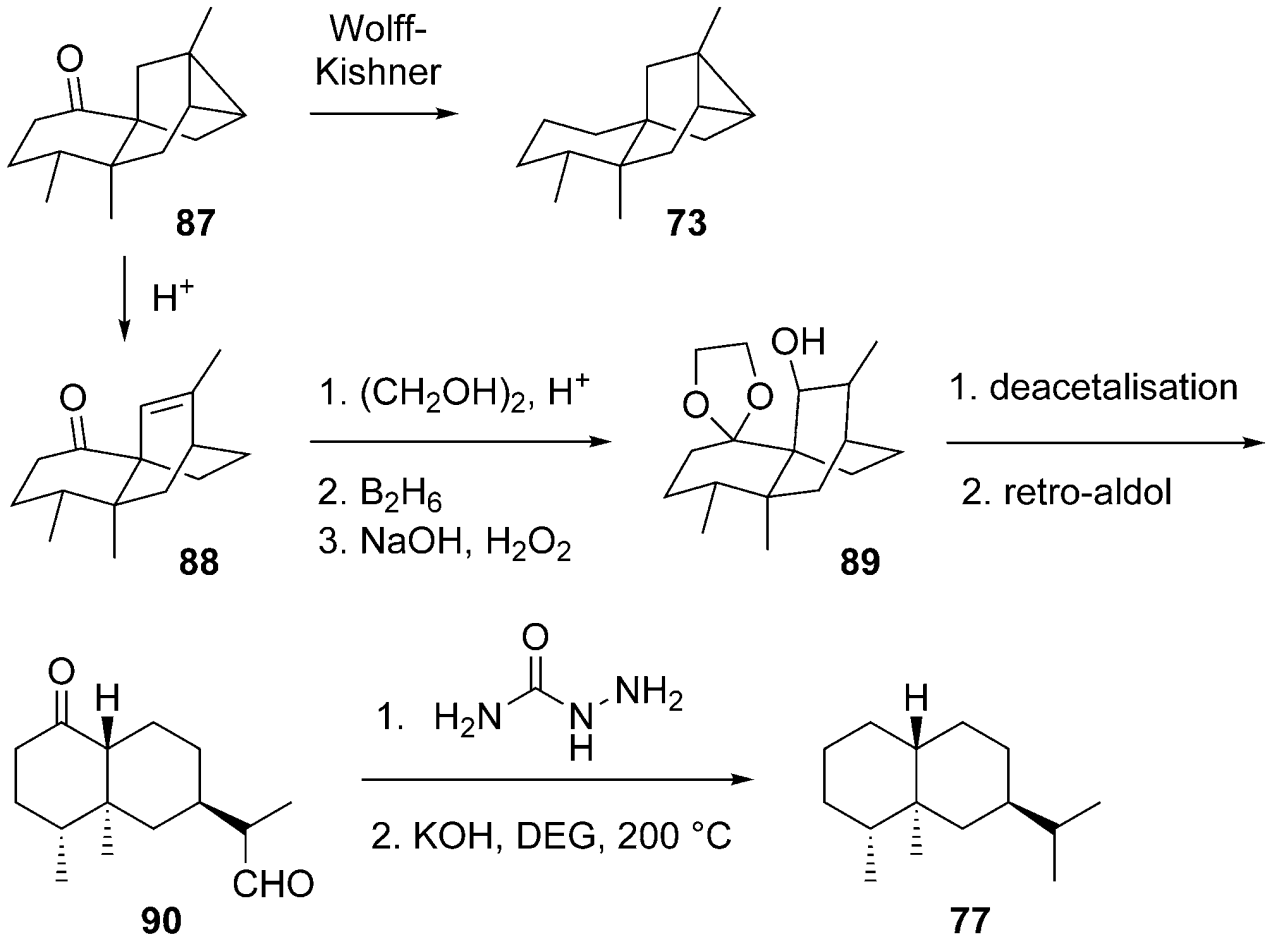
Chemical correlation of ishwarane (**73**) with nootkatane (**77**).

Valencene hydrate (**71**), arising from **H2 b** by attack of water, has been isolated from orange juice. For comparison this compound and its C10 epimer **72** were synthesised from **68** by epoxidation and epoxide opening with LiAlH_4_. Unfortunately, no optical rotations were given, but full ^13^C‐NMR data are available.^[197]^

(−)‐Ishwarane (**73**, [α]_D_=−40.33) was first isolated from *Aristolochia indica* where it co‐occurs with biosynthetically linked **70**.^[191]^ The compound has been chemically correlated through (+)‐ishwarone (**87**) that can be converted into **73** by Wolff–Kishner reduction (Scheme 22).^[191]^ Compound **87** undergoes ring opening to (−)‐isoishwarone (**88**) by treatment with acid.^[198]^ Its further conversion by acetalisation, hydroboration and oxidation leads to **89**, that upon deacetalisation and retro‐aldol reaction results in **90**. Reduction through the bis‐semicarbazone yields (+)‐nootkatane (**77**), thus firmly establishing the absolute configuration of **73**.^[199]^ Ishwarane was subsequently also found in many other plants,[^185^, ^200^, ^201^, ^202^, ^203^, ^204^] while 8,12‐*seco*‐ishwaran‐12‐ol (**74**, [α]_D_=−165, *c* 0.1, CHCl_3_) has only once been reported from *Litsea amara*.^[205]^ Its absolute configuration has not been formally established, but was suggested to correspond to that of **73**. Full ^13^C‐NMR data for **73** and **74** are available.[^206^, ^207^]

(−)‐Agarospirene (**75**) was first obtained by pyrolysis of the benzoate ester of agarospirol, a compound isolated from agarwood.^[207]^ Its structure has also been ascribed to a natural product isolated from the liverworts *Scapania robusta* and *Scapania maxima*,[^208^, ^209^] but a later synthesis of **75** ([α]_D_^22^=−11, *c* 0.3) and its stereoisomers demonstrated that the natural product was identical to (−)‐hinesene (**59**).^[178]^ Complete ^1^H‐ and ^13^C‐NMR data for **75** were reported.^[178]^

### 4.3 Rearranged eudesmanes from H3

**Scheme 23:**
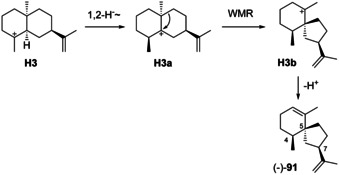
Rearranged eudesmanes from **H3**: spirovetivadiene (**91**).

Natural rearranged eudesmanes from **H3** are unknown. The only known compound is (4*S*,5*R*,7*R*)‐spirovetivadiene (**91**) that has been obtained by synthesis ([α]_D_^22^=−3, *c* 0.6). Its hypothetical biosynthesis from **H3** would require a 1,2‐hydride shift to **H3 a**, ring contraction to **H3 b** and deprotonation (Scheme 23). Full ^1^H‐ and ^13^C‐NMR data are available.^[178]^

### 4.4 Rearranged eudesmanes from H4

**Scheme 24:**
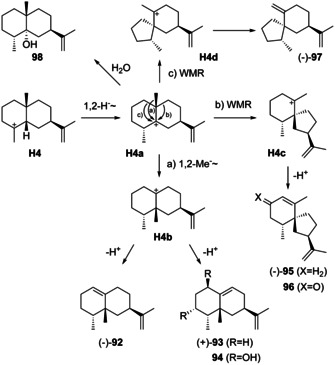
Biosynthesis of rearranged eudesmanes from **H4**.

Known rearranged eudesmanes from intermediate **H4** (Scheme 24) are represented by (−)‐4‐*epi*‐eremophilene (**92**), (+)‐5‐*epi*‐aristolochene (**93**), (−)‐premnaspirodiene (**95**, also named spirovetivene), (−)‐spirolepechinene (**96**) and 4βH,7αH,10β‐eudesm‐11‐en‐4α‐ol (**98**). The unusual sesquiterpene **97** requires a ring contraction to **H4 d** and deprotonation.

Both compounds **92** ([α]_D_^25^=−22.7, *c* 0.17, CHCl_3_) and **93** ([α]_D_^25^=+8.13, *c* 0.16, hexane) were obtained by synthesis from capsidiol (**94**).[^175^, ^210^] Notably, **93** is also the biosynthetic precursor to **94**,^[211]^ as was demonstrated by incubation of [1,1‐^3^H_2_]FPP with cell‐free enzyme preparations from *Nicotiana tabacum*, yielding radioactively labelled **93**. Furthermore, ^14^C‐labelled **93** was incorporated into **94** in feeding experiments with *N. tabacum* and *Capsicum annuum*.[^212^, ^213^] Subsequent work resulted in the purification of tobacco 5‐*epi*‐aristolochene synthase (TEAS),^[214]^ cloning of the genes from *N. tabacum* and *C. annuum* and expression in *Escherichia coli*,[^215^, ^216^, ^217^] and determination of the first crystal structure of a plant terpene synthase.^[218]^ Based on this structure the active site residue Tyr520 was suggested to be responsible for reprotonation of the intermediate (−)‐**1**. Consistent with this hypothesis, the Y520F enzyme variant gave (−)‐**1** as a single product.^[219]^ Also the 5‐*epi*‐aristolochene‐1,3‐dihydroxylase for the biosynthesis of **94** from **93** has been identified.^[220]^ For the biotechnological access to **93** the *epi*‐aristolochene synthase gene has been heterologously expressed in *E. coli*,^[221]^ in *Oryza sativa*,^[222]^ and in yeast in which optimisation of the strain and the culture conditions resulted in a high titre production.^[223]^ A thermostable variant of EAS has been created.^[224]^

Along similar lines of research, **95** has first been isolated from *Premna latifolia* ^[225]^ and subsequently from *Lepechinia bullata* ([α]_D_^20^=−88, *c* 0.501, CHCl_3_) in which it co‐occurs with **97** ([α]_D_^20^=−32, *c* 0.125, CHCl_3_).^[226]^ The premnaspirodiene synthase (also known as vetispirodiene synthase) from *Hyoscyamus muticus* (HPS) has been characterised.[^227^, ^228^] Another sesquiterpene synthase (Tps32) from *Solanum lycopersicum* with 90 % sequence identity to HPS was initially described as viridiflorene synthase,^[229]^ but a later study showed that Tps32 is indeed active as premnospiradiene synthase.^[230]^ Compound **95** is the parent hydrocarbon of (−)‐solavetivone (**96**),[^231^, ^232^] for which a premnaspirodiene oxygenase was reported.^[233]^

A detailed analysis of the product profiles of TEAS and HPS has led to the characterisation of several side products and demonstrated that TEAS produces minor amounts of **95**,^[234]^ while HPS generates small quantities of **93** from FPP.^[235]^ Domain swapping experiments between TEAS and HPS resulted in enzyme variants making mixtures of **93** and **95** and allowed the identification of domains that conferred specificity for these two products.^[236]^ After the crystal structure of TEAS had become available, a systematic and rational approach targeting nine selected residues within and near the active site in all 2^9^=512 combinations for a functional interconversion between TEAS and HPS was surveyed.[^237^, ^238^] Finally, compound **98** has been isolated from orange juice. ^1^H‐ and ^13^C‐NMR data for **92**,^[175]^ **93**,^[210]^ **95**,[^178^, ^226^] **97**,^[226]^ and **98** ^[197]^ have been published.

### 4.5 Rearranged eudesmanes from H5

**Scheme 25:**
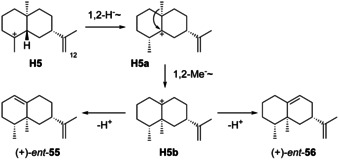
Biosynthesis of rearranged eudesmanes from **H5**.

Only a few reports about rearranged eudesmanes from **H5** from Nature are available (Scheme 25). Terpene synthases for *ent*‐**55** have been characterised from the myxobacterium *Sorangium cellulosum* ([α]_D_^25^=+131.7, *c* 1.0, CHCl_3_)^[239]^ and the plant pathogenic fungus *Fusarium fujikuroi*.^[240]^ The cyclisation mechanism of (+)‐eremophilene synthase from *F. fujikuroi* was studied by isotopic labelling experiments that showed selective deprotonation from C12 of FPP in the formation of the intermediate (−)‐**1**, allowed to follow the 1,2‐hydride shift from **H5** to **H5 a**, and demonstrated that the final deprotonation from **H5 b** to *ent*‐**55** proceeds with loss of the same proton as incorporated in the cyclisation of (−)‐**1** to **H5** (Scheme 7).^[240]^ A crystal structure of *ent*‐**55** ^[239]^ and full NMR data assignments have been published.[^239^, ^240^] Only a synthetic study towards *ent*‐**56** ([α]_D_^25^=+12.5, *c* 2.5, CHCl_3_) is available.^[241]^

### 4.6 Rearranged eudesmanes from H6

**Scheme 26:**
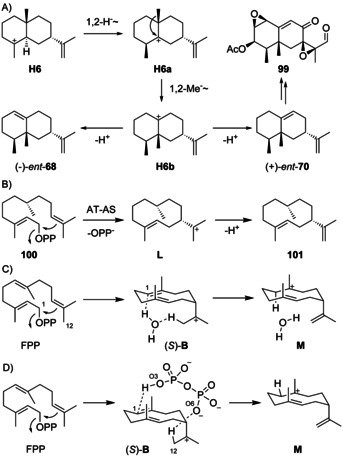
A) Biosynthesis of rearranged eudesmanes from **H6**. B) Cyclisation of (*R*)‐5,6‐dihydro‐FPP (**100**) to **101** by AT‐AS. C) Proposed water‐mediated proton transfer from (*S*)‐**B** to **M** in the biosynthesis of *ent*‐**70**.

Rearranged molecules from **H6** (Scheme 26 A) are (−)‐valencene (*ent*‐**68**) and (+)‐aristolochene (*ent*‐**70**) that has been isolated from *Aspergillus terreus* ([α]_D_=+79.4, *c* 0.0176, hexane),[^192^, ^196^] and *Penicillium roqueforti*, in which it occurs together with **2**.[^148^, ^242^, ^243^] The absolute configuration has been established by synthesis of (−)‐**70** from (+)‐valencene (**68**).^[192]^ (+)‐Aristolochene synthase was first isolated from *P. roqueforti* (PR‐AS)^[244]^ and is also present in *A. terreus* (AT‐AS).^[245]^ Subsequent gene cloning and expression gave efficient access to the recombinant enzymes.[^246^, ^247^] A biphasic flow reactor system for the biocatalytic production of *ent*‐**70** has been developed.^[248]^

Notably, PR‐AS produces a mixture of *ent*‐**70** as the main and *ent*‐**68** and (−)‐**1** as side products, while AT‐AS yields *ent*‐**70** as a single product.[^249^, ^250^] Isotopic labelling experiments demonstrated that the cyclisation of FPP to *ent*‐**70** proceeds with inversion of configuration at C1 and the specific loss of a proton from C12.^[245]^ The E252Q variant of PR‐AS yielded (−)‐germacrene A (**1**) as the only product.^[250]^ Further support of (−)‐**1** as an intermediate was obtained by the observed cyclisation of (*R*)‐5,6‐dihydro‐FPP (**100**) to the germacrene A analogue **101** by AT‐AS (Scheme 26 B).^[251]^ Similar experiments have been carried out with fluorinated FPP analogues.[^252^, ^253^] On the other hand, instead of a true pathway intermediate, (−)‐**1** could only be a shunt product. Allemann and co‐workers have argued for this view, as (−)‐**1** was not accepted as a substrate by PR‐AS,^[249]^ and a computational study showed feasibility of a water‐mediated direct proton transfer from (*S*)‐**B** to **M** that could further cyclise to **H6** (Scheme 26 C).^[254]^ However, the same workers later excluded this possibility experimentally, because the incorporation of deuterium from D_2_O at C1 of *ent*‐**70** proceeded with *Re* face attack.^[255]^ Based on the crystal structure of PR‐AS the active site residue Tyr92 was suggested to serve as a general acid in the reprotonation of (−)‐**1**,^[256]^ but also this hypothesis was disfavoured by site‐directed mutagenesis.^[250]^ A more detailed picture was subsequently obtained by the crystal structure of AT‐AS, providing evidence that the diphosphate anion is ideally positioned to act as a general acid and base relevant for i) the deprotonation of (*S*)‐**B**, with the proton taken up by O6, and ii) the reprotonation of the resulting (−)‐**1** with donation of a different proton from O3 (this process may also be concerted with **1** as a highly transient species, Scheme 26 D).^[257]^ The results of a site‐directed mutagenesis suggest that the thus formed eudesmane cation **H6** is stabilised by W334 of PR‐AS or W308 of AT‐AS.^[258]^ Cationic aza‐analogues of **H6** have been shown to efficiently inhibit catalysis by PR‐AS.[^259^, ^260^]

The sesquiterpene hydrocarbon *ent*‐**70** is the biosynthetic precursor to PR toxin (**99**),^[261]^ a potent mycotoxin that targets transcription and protein biosynthesis with a lethal dose of LD_50_=5 mg kg^−1^ in mice,[^262^, ^263^, ^264^] and a series of other oxidation products that are likely pathway intermediates.[^265^, ^266^, ^267^, ^268^, ^269^] Surprisingly, despite the potential of mycotoxin biosynthesis *P. roqueforti* is traditionally used for the production of blue cheese, which is explainable by the rapid degradation of **99** under cheese fermentation conditions.^[270]^ Biosynthetic hypotheses linking these oxidised metabolites have been investigated by feeding of labelled precursors[^148^, ^269^] and discussed on the grounds of the biosynthetic gene cluster,[^271^, ^272^, ^273^] but apart from the aristolochene synthase and the poorly characterised eremofortin C oxidase^[274]^ for the installation of the aldehyde function in **99** little is known about the enzymes involved in fungal toxin biosynthesis.

## 5 Guaianes

### 5.1 Guaianes formed by C4 protonation of germacrene A

**Scheme 27:**
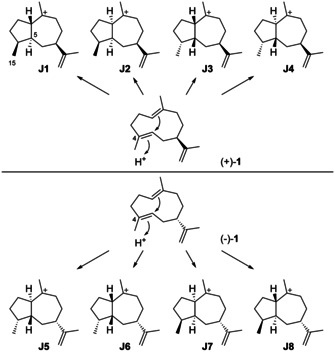
Cyclisations induced by reprotonation of **1** at C4 to **J1**–**J8**.

Eight cationic intermediates can be formed from the enantiomers of **1** by protonation at C4 and ring closure (Scheme 27). These cations exhibit four stereogenic centres, leading to a maximum number of 2^4^=16 possible stereoisomers, but two of the stereogenic centres are not set independently, since the C4/C5 double bond in **1** is *E*‐configured and the ring closure proceeds by *anti* addition, that is, Me15 and H5 must be arranged *trans*. Thus, only eight stereoisomers are relevant to this pathway, namely **J1**–**J4** from (+)‐**1**, and their enantiomers **J5**–**J8** from (−)‐**1**.

### 5.2 Guaianes formed from cations J1 and J2

**Scheme 28:**
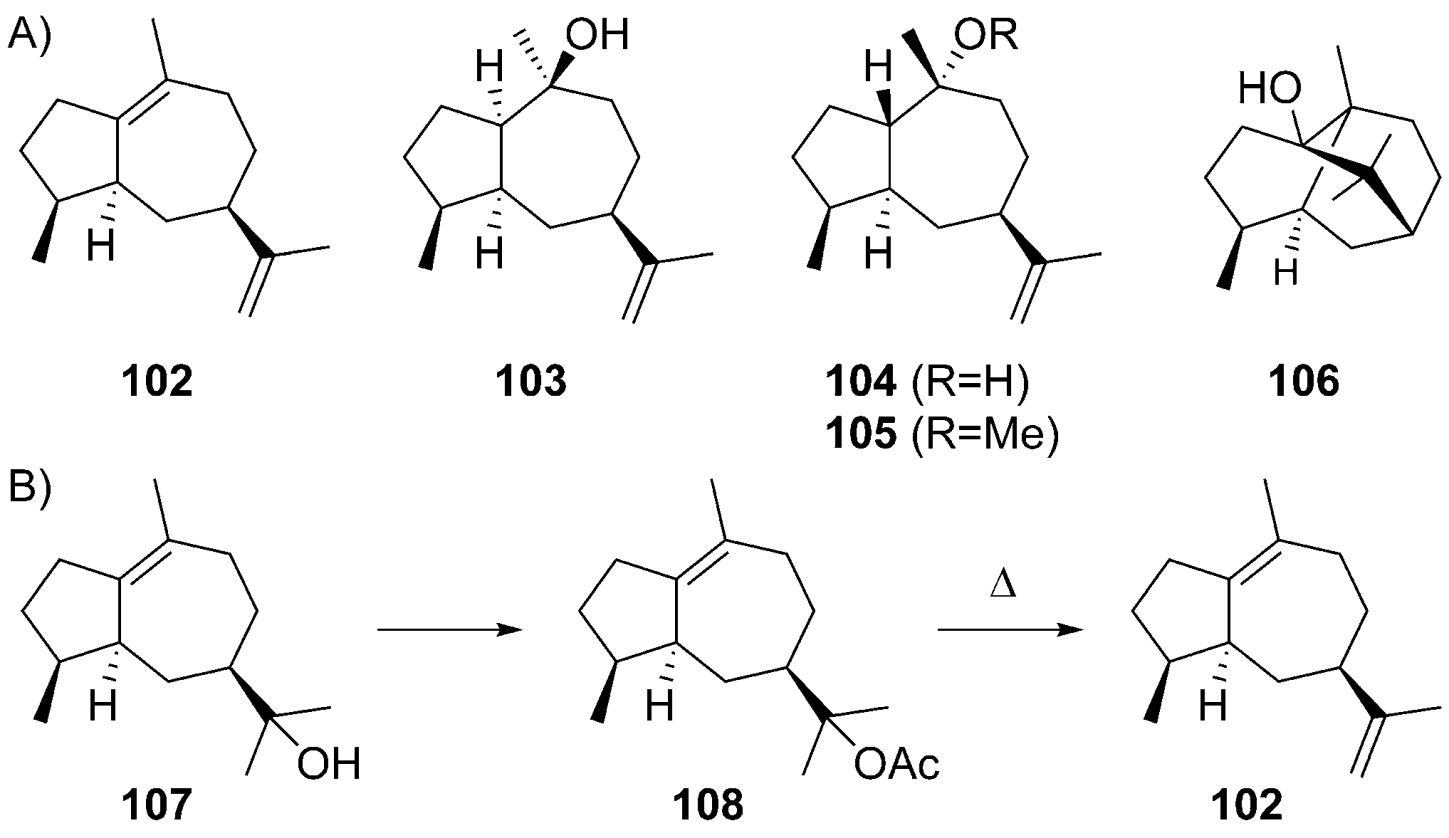
A) Guaianes derived from **J1** and **J2**, initially reported structures of pogostol (**104**) and pogostol methyl ether (**105**), and patchoulol (**106**). B) Synthesis of **102** from bulnesol (**107**).

Guaianes from cations **J1** and **J2** include δ‐guaiene (**102**) and pogostol (**103**, Scheme 28 A). δ‐Guaiene is also named α‐bulnesene and can in principle be generated by the deprotonation of **J1** or **J2**, while **103** derives from **J2** by *Si* face attack of water. Compound **102** was first isolated from the patchouli oil of *Pogostemon cablin* and given its premier name δ‐guaiene in 1950. Initially, only the planar structure with insecure positioning of double bonds was determined, with a reported optical rotation close to zero of [α]_D_=+0.32.^[275]^ Later, bulnesol (**107**) was chemically converted into **102** by pyrolysis of its acetate **108** (Scheme 28 B), leading to a material with an [α]_D_=0,^[276]^ that was thus inconclusive for assigning the absolute configuration of **102** from the fully established structure of **106**.[^277^, ^278^] Because **102** is accompanied by patchouli alcohol (**106**) in *P. cablin*, it was suggested that both compounds should have coinciding absolute configurations, but at this time for **106** still a wrong structure was assumed (vide infra).^[276]^ A subsequent stereoselective synthesis from α‐cyperone (**16**, Scheme 9) and comparison of the optical rotatory dispersion (o.r.d.) curves of synthetic and natural **102** finally established its structure.[^279^, ^280^] Compound **102** is known from several other plants[^281^, ^282^, ^283^, ^284^] including *Piper fimbriulatum*,^[285]^ in which it occurs together with **2**. In addition, **102** can be produced by cultured cells from *Aquilaria crassna* and *Aquilaria sinensis*,[^286^, ^287^] resulting in the discovery of the δ‐guaiene synthase from *A. crassna*.^[288]^ Compound **102** is also one of the main products of the α‐guaiene synthase from *V. vinifera* ^[289]^ and a side product of the patchoulol synthase from *P. cablin*.[^22^, ^290^] The complete ^1^H‐ and ^13^C‐NMR data of **102** are available.^[286]^

Pogostol (**103**) was first isolated from *P. cablin* ([α]_D_=−20.2, *c* 8.7).^[291]^ Since then, **103** was reported from various other plant sources[^292^, ^293^, ^294^, ^295^, ^296^] and is known from the fungus *Geniculosporium*.^[297]^ A relative configuration was first assigned for pogostol O‐methyl ether (**105**) from *Artabotrys stenopetalus*,^[298]^ followed by the assignment of the relative configuration of **104** for pogostol by Weyerstahl and co‐workers.^[293]^ A subsequent synthesis of the reported structures **104** and **105** for pogostol and its methyl ether demonstrated that both assignments were erroneous.^[299]^ Amand et al. then gave a correction as **103**.^[295]^ Although pogostol is long known and fairly widespread in Nature, the absolute configuration still remains to be determined. For unclear reasons the structure of *ent*‐**103** has been assigned to the CAS number of pogostol (21698‐41‐9), while in fact **103** may be more likely, because this corresponds to the main product **106** of the patchoulol synthase from *P. cablin* that also makes **103** as a side product.^[22]^ ^1^H‐ and ^13^C‐NMR data of **103** are reported in the literature.[^292^, ^293^, ^294^, ^295^, ^297^]

**Scheme 29:**
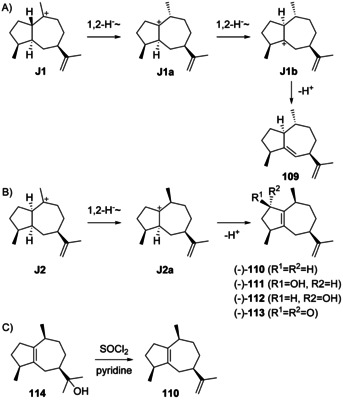
Biosynthesis of guaianes from A) **J1** and B) **J2**. C) Chemical correlation of **110** with guaiol (**114**).

The sesquiterpene 1,4‐*diepi*‐γ‐gurjunene (**109**, Scheme 29 A) was isolated from the sponge *Cymbastela hooperi* ([α]_D_= +34.6, *c* 0.11, CHCl_3_).^[300]^ The formation of this compound can be understood from **J1** by two sequential 1,2‐hydride shifts via **J1 a** to **J1 b** and deprotonation. Since the absolute configuration of **109** has not been determined, it may also be derived from intermediate **J5**. Full ^1^H‐ and ^13^C‐NMR data have been provided for **109**.^[300]^

α‐Guaiene (**110**, Scheme 29 B) may instead arise from **J2** by 1,2‐hydride migration to **J2 a** and deprotonation. It is the universal precursor leading under simple aerial oxidation conditions to many fragrant volatiles of industrial importance such as (*R*)‐ and (*S*)‐rotundols (**111** and **112**) and rotundone (**113**) that exhibit a pleasant peppery or woody aroma.[^301^, ^302^, ^303^] Compound **110** ([α]_D_^19^=−64.5, *c* 3.584, dioxane) was initially obtained by dehydration of guaiol (**114**, Scheme 29 C).^[304]^ With the absolute configuration of **114** being specified,^[305]^ the full structure of compound **110** was also affirmed. Natural sources of **110** include several plant species[^63^, ^284^, ^285^, ^306^, ^307^, ^308^, ^309^, ^310^] and cell cultures from *Aquilaria crassna* and *A. sinensis*.[^286^, ^287^] A recombinant α‐guaiene synthase has been reported from *V. vinifera*,^[289]^ and **110** is also a side product of δ‐guaiene synthase from *A. crassa* ^[288]^ and patchoulol synthase from *P. cablin*.[^22^, ^290^] The biosynthesis of **110** is also possible from **K1** (Scheme 32, Section 5.5) by 1,2‐hydride shift and deprotonation, but the co‐occurrence with **102** in several species,[^284^, ^285^, ^286^, ^287^, ^307^, ^308^] whose formation can best be understood from **J1** or **J2**, together with the observation of both compounds in the product profiles of several terpene synthases[^22^, ^288^, ^289^, ^290^] speaks in favour of a common biosynthesis through **J2**. Full ^1^H‐ and ^13^C‐NMR data of **110** are provided.[^284^, ^308^]

### 5.3 Guaianes formed from cations J3 and J4

**Scheme 30:**
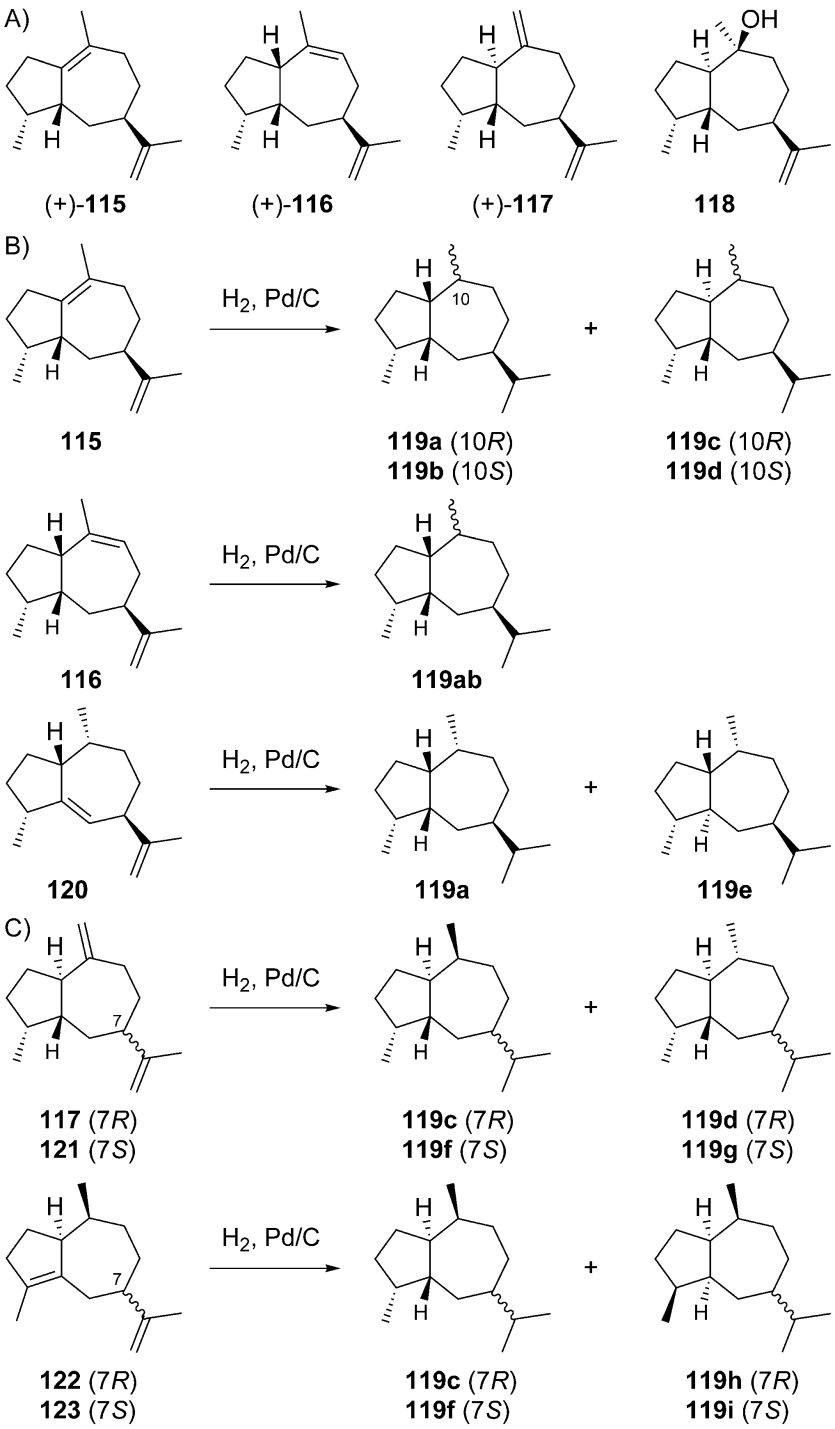
A) Structures of **115**–**118**. Correlations through hydrogenation products B) of **115** and **116** to **120** and C) of revised **117** to aciphyllene (**122**, see text).

Guaianes from **J3** and **J4** include guaia‐1(10),11‐diene (**115**) that is accessible through both cations by deprotonation, and guaia‐9,11‐diene (**116**) obtainable by loss of a proton from **J3** (Scheme 30 A). Deprotonation of **J4** can lead to guaia‐10(14),11‐diene (**117**), a compound for which we revise the structure here based on the reason given below, while the attack of water to **J4** can give 4,5‐*diepi*‐pogostol (**118**). For **118** this discussion is hypothetical, because this compound was only obtained in racemic form by synthesis and is not known as natural product.^[299]^

The hydrocarbons (+)‐**115** and (+)‐**116** were both isolated only from the fruits of *Peucedanum tauricum*.^[311]^ Their co‐occurrence in one organism suggests that they may have the same cationic precursor **J3**. The absolute configurations of **115** and **116** were specified by comparison of their hydrogenation products to those obtained from (+)‐γ‐gurjunene (**120**, Scheme 30 B),^[312]^ leading to one common product (**119 a**) from all three materials, as judged by GC analysis using two different chiral stationary phases.

Guaia‐10(14),11‐diene (**117**) is only known from *Abies koreana*.^[121]^ Its absolute configuration was elaborated using the same hydrogenation strategy as for **115** and **116** with chemical correlation to aciphyllene (**122**, Scheme 30 C). At the stage of this work the structure of **123** with 7*S* stereochemistry was assigned for aciphyllene,^[284]^ which would have led to the hydrogenation products **119 f** and **119 i**, and therefore the structure of **121** was concluded for the natural product from *A. koreana* expected to give the hydrogenation products **119 f** and **119 g**. However, shortly after the structure of aciphyllene underwent a revision to (7*R*)‐**122**.^[313]^ In conclusion, the truly obtained hydrogenation products from aciphyllene were **119 c** and **119 h**, with the consequence that the natural product from *A. koreana* must be revised herewith to **117**, expected to give **119 c** and **119 d**.

**Scheme 31:**
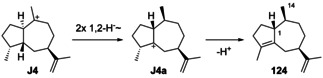
Hypothetical biosynthesis of 1‐*epi*‐aciphyllene (**124**).

**Scheme 32:**
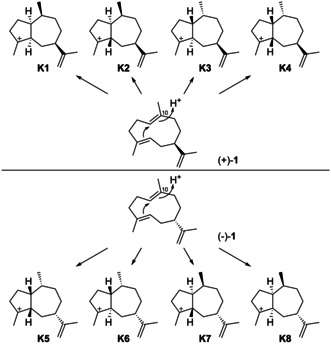
Cyclisations induced by reprotonation of **1** at C10 to **K1**–**K8**.

The synthetic compound 1‐*epi*‐aciphyllene (**124**) has been prepared from guaiol (**114**),^[314]^ but has not been discovered from Nature so far. Indeed, its biosynthesis is not easily understood, as its formation through the **K** series (Scheme 32, Secion 5.5) of cations cannot lead to a *cis*‐orientation of H1 and Me14. If **124** exists at all as a natural product, two sequential 1,2‐hydride migrations from **J4** to **J4 a** and deprotonation could explain its formation (Scheme 31). Full ^1^H‐ and ^13^C‐NMR data for **124** were reported,^[314]^ but unfortunately no optical rotation that would be useful for comparison in case of its future isolation.

### 5.4 Guaianes formed from cations J5–J8

Despite the fact that for **103** the absolute configuration has not been determined and this compound could in principle arise through **J6**, no guaianes from **J5**–**J8** are known. The absolute configuration of 1,4‐*diepi*‐γ‐gurjunene (**109**) from *C. hooperi* would be most interesting to know, as sponges may produce the optical antipodes of plant compounds.

### 5.5 Guaianes formed by C10 protonation of germacrene A

Considering the discussion above, there are also only four logical cationic intermediates (**K1**–**K4**) after the cyclisation from (+)‐**1** initiated by C10 protonation (Scheme 32). Likewise, (−)‐**1** can produce four additional candidates (**K5**–**K8**).

### 5.6 Guaianes formed from cations K1 and K2

**Scheme 33:**
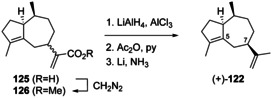
Chemical correlation of “ciphyllic acid” to **122** (corrected structure).

A deprotonation from C5 of K1 or K2 provides aciphyllene (122), also named guaia‐4,11‐diene. Compound **122** was first isolated from *Lindera glauca* in 1983 ([α]_D_^20^=+153.0).^[284]^ Its structure was erroneously elucidated by Kubota et al. as that of 7‐*epi*‐aciphyllene (**123**) by chemical correlation with aciphyllic acid (**125**, Scheme 33),[^284^, ^315^] a compound that had been reported with 7*S* configuration.^[316]^ The structure was later corrected to **122** by synthesis from (+)‐dihydrocarvone (**15**).^[313]^ Whether this means that also **125** should be revised to have 7*R* configuration or the material had undergone epimerisation at C7 during the transformations into **122** remains unclear at this stage. However, since Kubota and co‐workers^[315]^ as well as Liu and Yu^[317]^ have reported different NMR data for “aciphyllic acid”, in both cases with 7*S* configuration, at least one of these structures must be wrong. Thus it may be likely that the Japanese workers have indeed started their correlation of “aciphyllic acid” to **122** from a material with 7*R* configuration. (+)‐Aciphyllene (**122**) was later also found in *Dumortiera hirusta*,^[153]^ and with undetermined absolute configuration from the essential oil of *Xylopia rubescens*.^[310]^ It is also known as a side product of the recombinant patchoulol synthase from *Pogostemon cablin*,^[290]^ a multi‐product terpene synthase for which all products retain the (7*R*) stereochemistry introduced in the intermediate (+)‐1 and thus further supporting the structural reassignment for **122**. Moreover, total syntheses from (*R*)‐limonene by Srikrishna et al.^[318]^ and from guaiol (**114**) by Huang et al.^[314]^ were conducted. The ^1^H‐ and ^13^C‐NMR data of **122** have been published.[^153^, ^284^]

### 5.7 Guaianes formed from cations K3 and K4

**Scheme 34:**
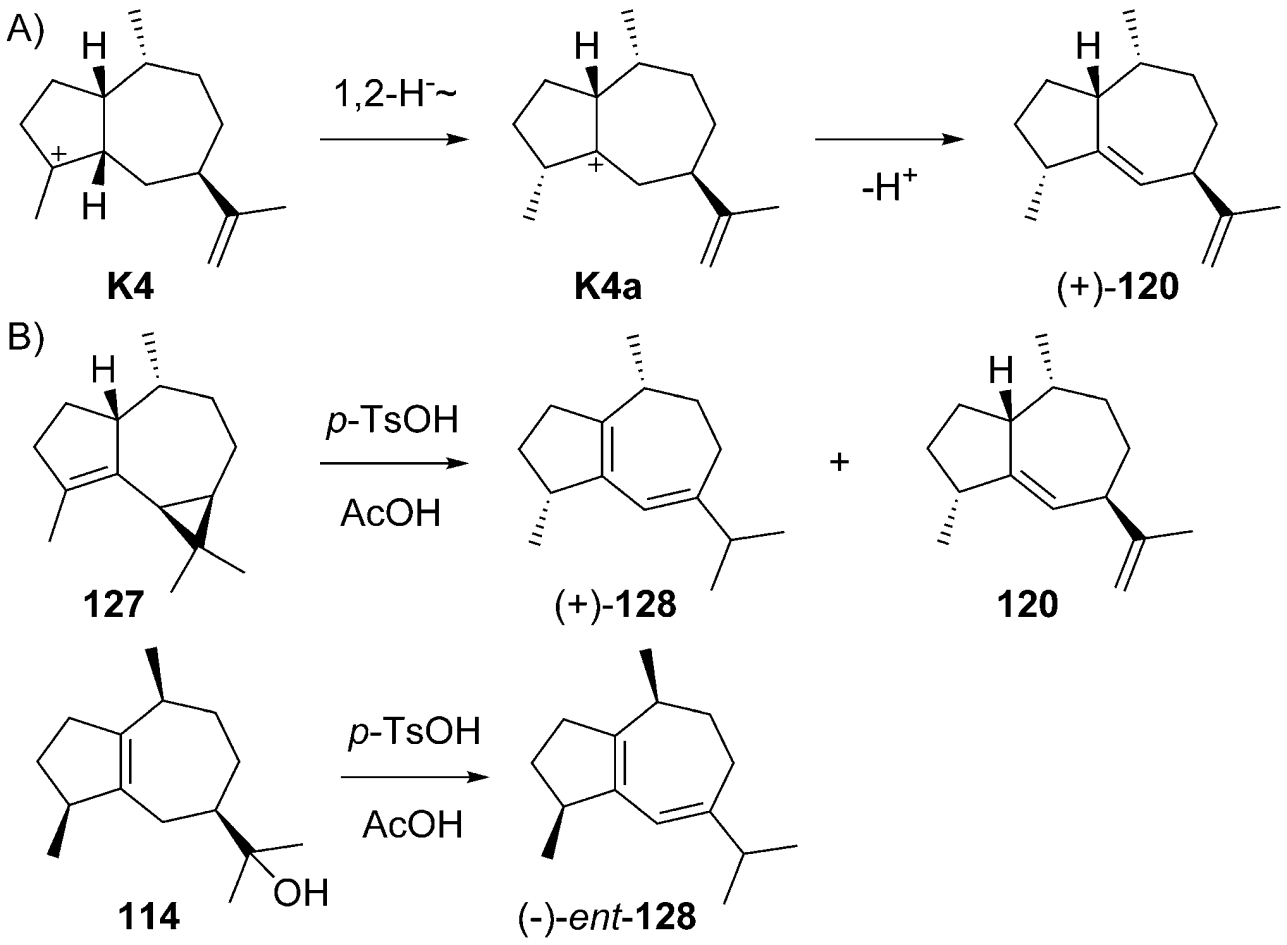
A) Biosynthesis of **120**. B) Correlation of **120** with **127** and **114**.

One of the most important sesquiterpenes derived from the **K** series is (+)‐γ‐gurjunene (**120**). Its formation can be understood from **K4** by 1,2‐hydride shift to **K4 a** and deprotonation (Scheme 34 A). This component was first discovered from the gurjun balsams of several species of *Dipterocarpus* ([α]_D_= +147, CHCl_3_).[^314^, ^319^] Its absolute configuration was illuminated by correlation with α‐gurjunene (**127**) and guaiol (**114**, Scheme 34 B).^[312]^ While treatment of **127** with acid gave the isomerisation products (+)‐**128** and **120** identical to natural (+)‐γ‐gurjunene, the isomerisation of **114** produced (−)‐*ent*‐**128**. Compound **120** was also isolated from *Persea gamblei*.^[320]^ Complete ^1^H‐ and ^13^C‐NMR data have been published.[^300^, ^319^, ^321^]

**Figure 2:**
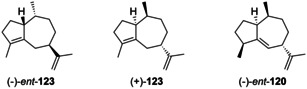
Structures of synthetic compounds *ent*‐**123**, **123** and *ent*‐**120**.

Compound (−)‐*ent*‐**123** (Figure 2) is only known as a synthetic material ([α]_D_^24^=−13.2, *c* 0.35, CHCl_3_) and could, as a hypothetical natural product, arise from **K3** or **K4** by deprotonation. It is wrongly presented in the synthesis paper that corrects the structure of (+)‐aciphyllene (**122**) as the assigned structure of this natural product (**123**, Scheme 30), while it represents in fact its enantiomer. Full ^1^H‐ and ^13^C‐NMR data are available.^[313]^

### 5.8 Guaianes formed from cations K5–K8

Natural products from the cations **K5**–**K8** are unknown. Synthetic compounds (Figure 2) include (+)‐7‐*epi*‐aciphyllene (**123**) obtained from (*R*)‐limonene ([α]_D_^27^=+13.5, *c* 1.3, CHCl_3_),^[318]^ and (−)‐γ‐gurjunene (*ent*‐**120**) made accessible through an enantioselective Morita‐Baylis–Hillman reaction using an enantiopure phosphine catalyst ([α]_D_^20^=−121.1, *c* 0.1 CHCl_3_).^[322]^ For both compounds full NMR data were provided.[^318^, ^322^]

## 6 Cyclised and Rearranged Guaianes

Further cyclisations eventually with skeletal rearrangements are important for two groups of compounds originating from **J1** and **J3**, while no examples from the other cations of the **J** series or from cations of the **K** series are known.

### 6.1 Compounds from J1

**Scheme 35:**
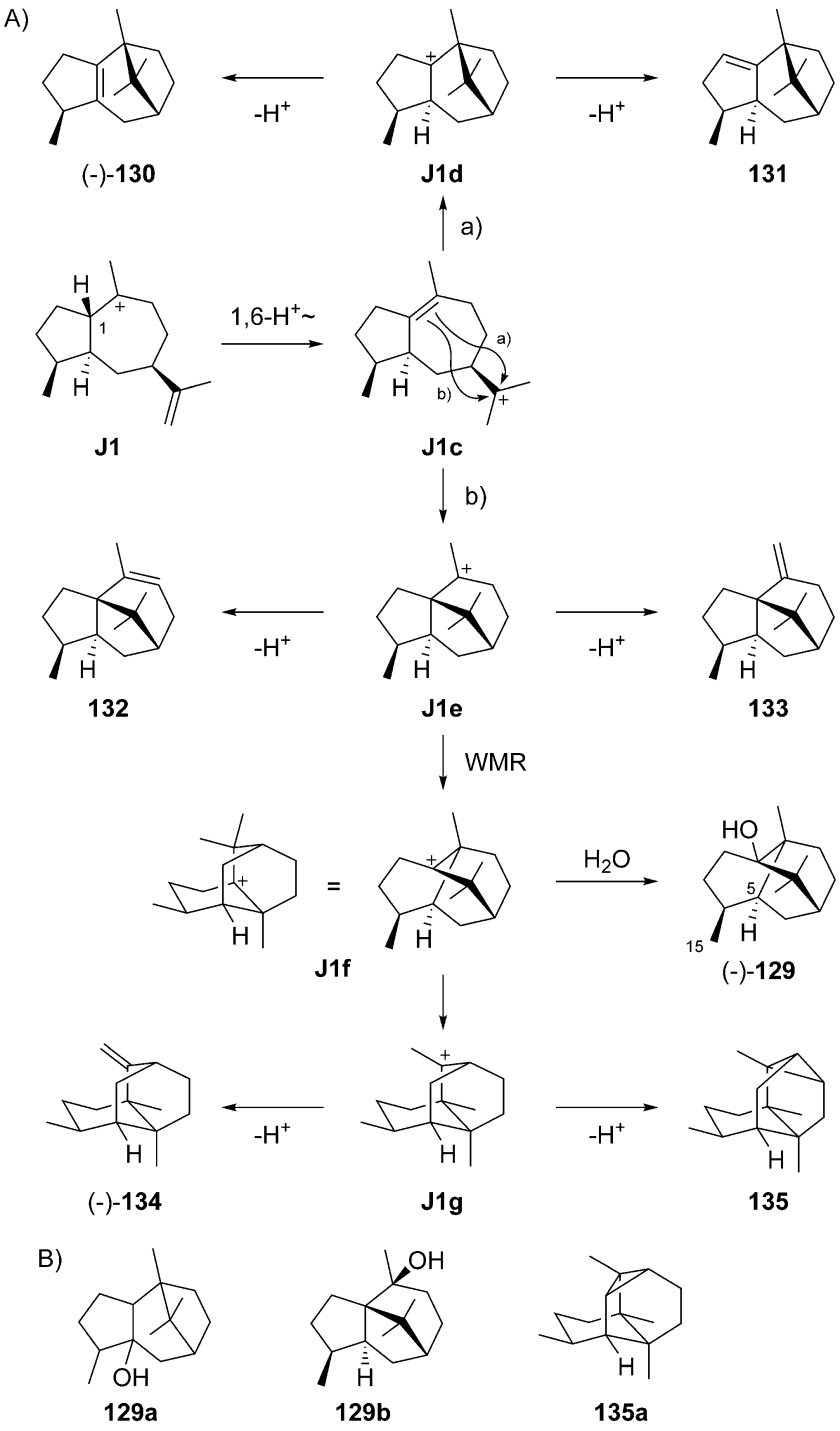
A) Biosynthesis of cyclised and rearranged guaianes from **J1**. B) Initially assigned structures for (−)‐patchouli alcohol (**129 a** and **129 b**) and cycloseychellene (**135 a**).

Compounds from **J1** include patchouli alcohol (**129**), the patchoulenes **130**–**133** and seychellenes **134** and **135** (Scheme 35 A). The common biosynthesis of these compounds can be understood from **J1** by a long range proton shift from C1 into the isopropenyl group to **J1 c**, followed by cyclisation to **J1 d** (path a) and deprotonation to β‐patchoulene (**130**) and δ‐patchoulene (**131**). An alternative cyclisation from **J1 c** to **J1 d** (path b) and deprotonation yields α‐patchoulene (**132**) and γ‐patchoulene (**133**). A Wagner–Meerwein rearrangement of **J1 e** to **J1 f** gives access to patchouli alcohol (**129**) by attack of water, while a methyl group migration to **J1 g** and deprotonation results in seychellene (**134**) or cycloseychellene (**135**). This pathway is in agreement with feeding experiments using radioactively labelled (4*R*)‐[2–^14^C,4‐^3^H]mevalonic acid,[^323^, ^324^] and with deuterium incorporation from (2‐^2^H)FPP at C5 of **129** and several side products from patchoulol synthase,[^22^, ^290^] while a reported additional deuteration at C15 is difficult to understand.

Patchouli alcohol or patchoulol (−)‐**129** was first isolated as the main constituent from patchouli oil (*P. cablin*) in 1869.^[325]^ The oil is one of the most important industrial fragrances that is widely used in perfumery and cosmetics products. Its planar structure was described more than 80 years later as that of **129 a** (Scheme 35 B).^[326]^ A structural revision based on chemical transformations and a synthesis from (+)‐camphor through **132** resulted in the assignment of structure **129 b**.[^327^, ^328^, ^329^] However, a subsequent X‐ray analysis of the chromic acid diester surprisingly led to the structure of **129**,^[330]^ suggesting that during the synthesis of this compound from **132** a similar skeletal rearrangement as in the biosynthesis must have taken place. A later synthesis from (*R*)‐carvone (*ent*‐38) resulted in (−)‐**129** ([α]_D_^25^=−121.3, *c* 2.3, CHCl_3_).^[331]^ Compound (−)‐**129** was also isolated from plants of the genera *Valeriana*[^332^, ^333^, ^334^] and *Nardostachys*[^335^, ^336^] The complete ^13^C NMR data of **129** are available.[^290^, ^333^, ^337^]

**Scheme 36:**
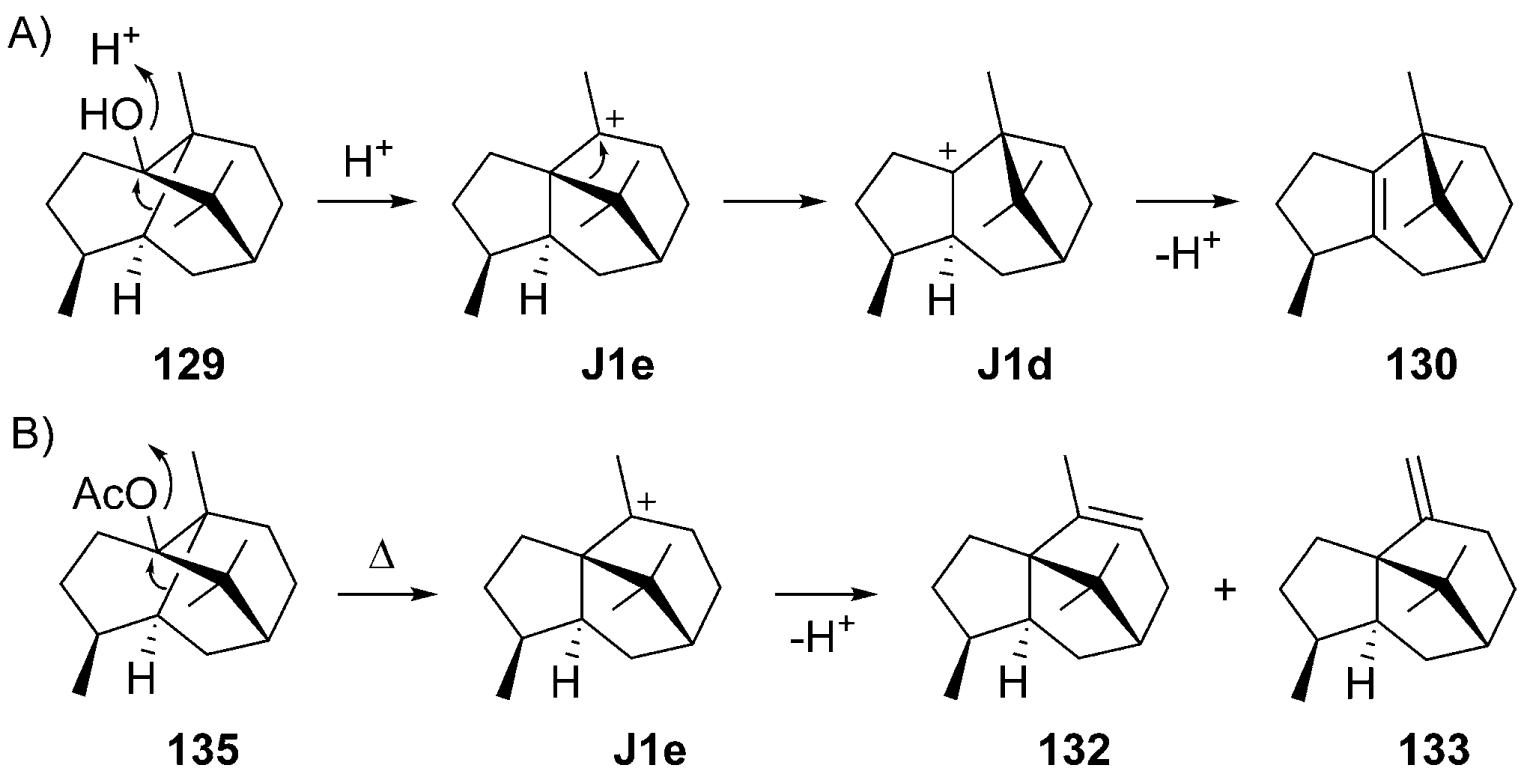
A) Acid promoted conversion of **129** into **130**. B) Pyrolysis of patchoulyl acetate (**135**) to patchoulenes **132** and **133**.

The patchoulenes **130–133** and seychellenes **134** and **135** have been reported to co‐occur with **129** in several species,[^307^, ^332^, ^334^, ^335^, ^336^, ^338^, ^339^] and also many of these compounds are observed as products of the patchoulol synthase,[^22^, ^290^] supporting their common biosynthesis through shared intermediates (Scheme 35 A) and corresponding absolute configurations. Formally, the absolute configuration of **130** ([α]_D_^30^=−42.6, *c* 10.51, CHCl_3_) was specified by chemical correlation with patchouli alcohol through acid treatment, at a time when **129 b** was believed to be the correct structure of this sesquiterpene alcohol. Pyrolysis of patchoulyl acetate (**135**) yielded a mixture of **132** and **133**, and dehydration with POCl_3_ resulted in a mixture of mainly **132** with **130** and **133**.^[328]^ A reinterpretation of the results from these experiments included a Wagner–Meerwein rearrangement (Scheme 36).^[340]^ Compound **131** was first obtained by the acid‐catalysed transformation of **129** ^[341]^ and later isolated from patchouli oil.^[342]^ The complete ^1^H and ^13^C NMR data of **130** are available,^[308]^ while those of **131–133** are lacking.

Seychellene (**134**, Scheme 35 A), [α]_D_=−72 (*c* 0.4, CHCl_3_),^[343]^ was first found in patchouli oil (“hydrocarbon G”),^[307]^ followed by structure elucidation through chemical degradation.[^340^, ^343^] A total synthesis of (−)‐**134** from (*R*)‐carvone (*ent*‐81) confirmed its absolute configuration.^[344]^ Cycloseychellene (**135**) was reported to possess the structure of **135 a** (Scheme 35 B) when it was first isolated from *P. cablin* in 1973.^[339]^ In 1981, Welch et al. synthesised (±)‐135 a and found that the spectral and chromatographic properties of the synthetic hydrocarbon differed significantly from those of the natural product.^[345]^ A re‐examination of the NMR spectra of cycloseychellene indicating that its structure should be corrected to that of **135**.^[346]^ The ^1^H‐ and ^13^C‐NMR data of 134 are available from the literature.[^308^, ^344^]

### 6.2 Compounds from J3

**Scheme 37:**
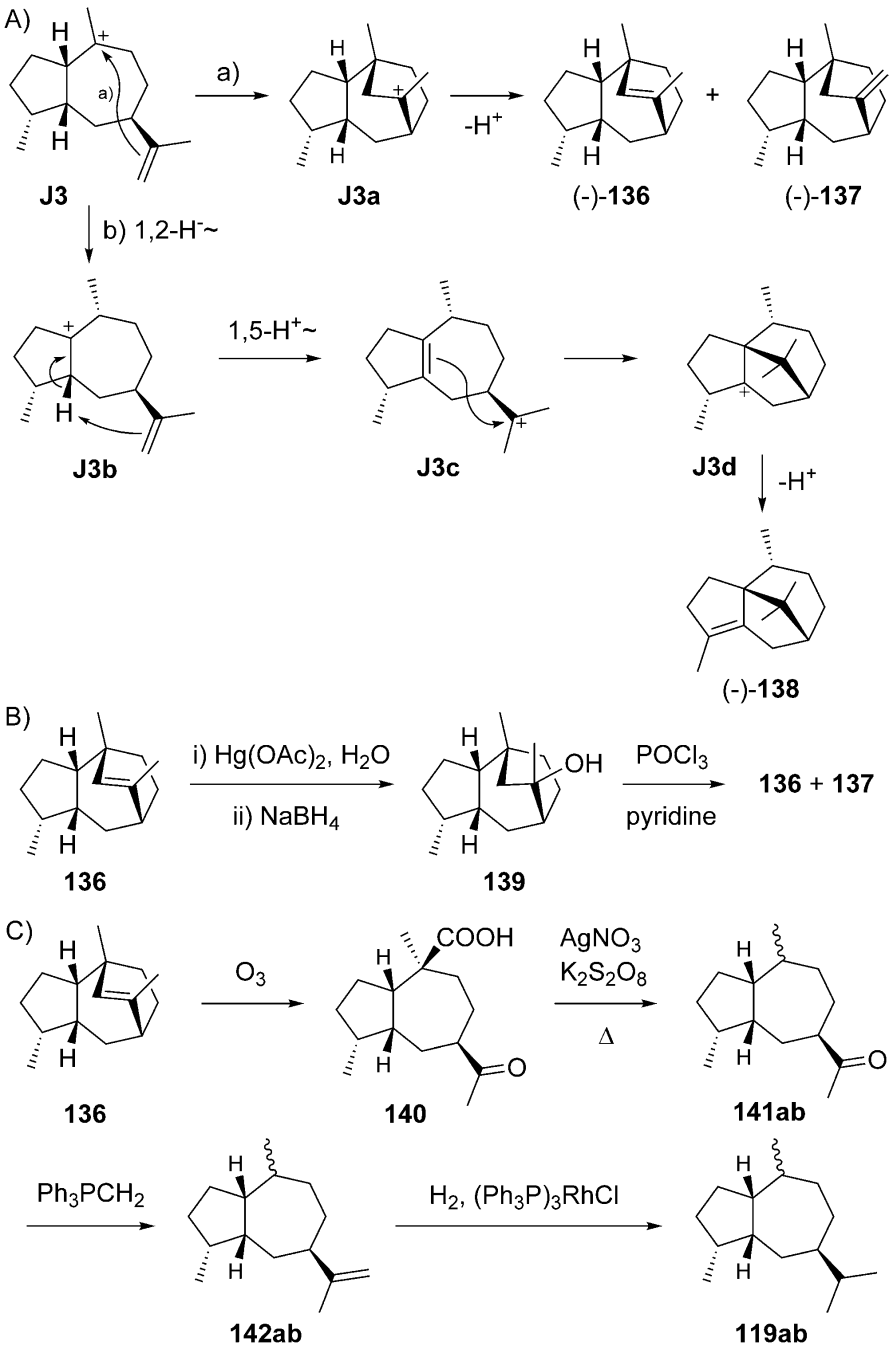
A) Biosynthesis of **136**–**138** from **J3**. Chemical correlation of B) **136** to **137**, and C) **136** to **119 a**, the hydrogenation product of (+)‐γ‐gurjunene.

The biosynthesis of rotundene (**136**), isorotundene (**137**) and cyperene (**138**) can be understood from **J3** (Scheme 37 A). Its cyclisation to **J3 a** (path a) and deprotonation yields **136** and **137**, while a 1,2‐hydride shift to **J3 b** (path b) followed by a 1,5‐proton shift to **J3 c**, cyclisation to **J3 d** and deprotonation result in **138**. This common biosynthetic pathway nicely explains the co‐occurrence of **136**–**138** in *Cyperus rotundus*.^[347]^ Compound **136** ([α]_D_=−16.3) was first reported from *C. rotundus* and *C. scariosus*,^[348]^ and later also from *C. alopecuroides*,^[349]^ but at this stage only with the planar structure. (−)‐Isorotundene (**137**) was isolated from *C. rotundus* whose relative configuration was determined by NOESY.^[347]^ This allowed to demonstrate that **136** has the same skeleton by conversion into rotundol (**139**) through oxymercuration and dehydration with POCl_3_ (Scheme 37 B). The absolute configuration of **136**, and thus also of **137**, was determined by ozonolysis to **140**, decarboxylation to a mixture of epimers **141 ab**, Wittig methylenation to **142 ab** and catalytic hydrogenation to **119 ab** (Scheme 37 C). One of these hydrocarbons was identical to **119 a** obtained by hydrogenation of **120** (Scheme 30 C). Complete ^1^H‐ and ^13^C‐NMR data for **137** have been reported,^[347]^ but are lacking for **136**.

The sesquiterpene **138** ([α]_D_^20^=−20.0, neat), was first isolated from *Cyperus rotundus*.[^350^, ^351^] Its absolute configuration was resolved by the chemical correlation through its hydrogenation product that was identical to a material derived from **129** by dehydration with POCl_3_ and hydrogenation.[^352^, ^353^] The (−)‐enantiomer of **138** was later isolated from several other plants.[^177^, ^349^, ^354^, ^355^, ^356^, ^357^, ^358^, ^359^, ^360^, ^361^, ^362^, ^363^, ^364^, ^365^, ^366^, ^367^] Full ^1^H‐ and ^13^C‐NMR data in CDCl_3_ and C_6_D_6_ have been reported.[^367^, ^368^]

## 7 Conclusions

Germacrene A shows a unique and interesting chemistry mainly characterised by its reactivity towards acid‐catalysed cyclisations and its thermal lability in a Cope rearrangement to β‐elemene. Similar observations have been made for other germacrenes,^[369]^ suggesting that the high ring strain associated with the 10‐membered ring in these systems may be a strong driving force for the observed reactions leading to much less strained compounds with 6‐membered rings. The reactivity built up by the ring strain is also used in enzymatic reactions towards sesquiterpenes for which germacrene A serves as an important intermediate. In enzyme reactions not only the formation of 6–6 bicyclic compounds, but also of 5–7 bicyclic derivatives can be achieved, and for both cases follow‐up chemistry by skeletal rearrangements can further increase the structural variability. Subsequent steps include oxidative and other modifications after terpene cyclisation, leading to numerous derivatives for each compound presented in this review, which further underlines the central importance of germacrene A in sesquiterpene biosynthesis.

## Conflict of interest

The authors declare no conflict of interest.

## Biographical Information

*Jeroen S. Dickschat studied Chemistry at TU Braunschweig and obtained his PhD in 2004. Since 2014 he is a Professor of Organic Chemistry and Biochemistry at the University of Bonn and holds a honorary Professorship at the NIOO Wageningen (The Netherlands). He also serves as an editor for the Beilstein Journal of Organic Chemistry. His research interests include the identification, biosynthesis and synthesis of microbial natural products, with a special focus on enzyme mechanisms*.

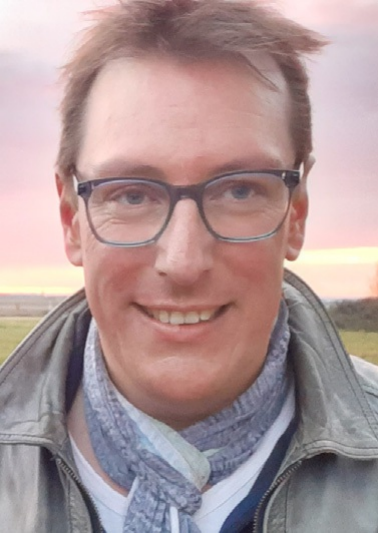

## Biographical Information

*Houchao Xu obstained his B.Sc. degree in Medicinal Chemistry from the Kunming Institute of Botany, Chinese Academy of Sciences. He joined the group of Prof. Dickschat at the University of Bonn in September 2019 to persue his PhD degree. His research focuses on the biosynthesis of bacterial terpenes and polyketides*.